# TIGR-Tas: A Family of Modular RNA-Guided DNA-Targeting Systems in Prokaryotes and Their Viruses

**DOI:** 10.1126/science.adv9789

**Published:** 2025-05-01

**Authors:** Guilhem Faure, Makoto Saito, Max E. Wilkinson, Natalia Quinones-Olvera, Peiyu Xu, Daniel Flam-Shepherd, Stephanie Kim, Nishith Reddy, Shiyou Zhu, Lilia Evgeniou, Eugene V. Koonin, Rhiannon K. Macrae, Feng Zhang

**Affiliations:** 1 Broad Institute of MIT and Harvard; Cambridge, USA.; 2 McGovern Institute for Brain Research at MIT; Cambridge, USA.; 3 Department of Brain and Cognitive Science, Massachusetts Institute of Technology; Cambridge, USA.; 4 Department of Biological Engineering, Massachusetts Institute of Technology; Cambridge, USA.; 5 Howard Hughes Medical Institute; Cambridge, USA.; 6 Department of Systems Biology, Harvard University; Boston, USA.; 7 National Center for Biotechnology Information, National Library of Medicine, National Institutes of Health, Bethesda, USA.

## Abstract

RNA-guided systems provide remarkable versatility, enabling diverse biological functions. Through iterative structural and sequence homology-based mining starting with a guide RNA-interaction domain of Cas9, we identified a family of RNA-guided DNA-targeting proteins in phage and parasitic bacteria. Each system consists of a Tandem Interspaced Guide RNA (TIGR) array and a TIGR-associated (Tas) protein containing a Nop domain, sometimes fused to HNH (TasH) or RuvC (TasR) nuclease domains. We show that TIGR arrays are processed into 36-nt RNAs (tigRNAs) that direct sequence-specific DNA binding through a tandem-spacer targeting mechanism. TasR can be reprogrammed for precise DNA cleavage, including in human cells. The structure of TasR reveals striking similarities to box C/D snoRNPs and IS110 RNA-guided transposases, providing insights into the evolution of diverse RNA-guided systems.

## One-Sentence Summary

A modular, programmable DNA-targeting system guided by tandem-spacer RNAs found in phage and parasitic bacteria enables human genome editing and reveals evolutionary connections to eukaryotic RNA-guided systems.

RNA-guided functional systems, in which a protein binds to a guide RNA that directs its activity to a complementary nucleic acid sequence, are inherently versatile molecular tools. In nature, RNA-guided systems allow a single enzyme to target multiple sequences depending on the loaded RNA guide. For example, in bacteria and archaea, CRISPR-Cas adaptive immune systems can recognize and cleave DNA or RNA of mobile genetic elements (MGEs) according to RNA guide sequences stored in a CRISPR array (1), and these arrays can be updated in response to new invaders (2). In eukaryotes, small RNAs such as microRNAs and PIWI-interacting RNAs (piRNAs) can direct regulatory responses upon their interaction with complementary target RNAs (3, 4). In eukaryotes and archaea, snoRNAs (small nucleolar RNAs) guide methylation and pseudouridylation of complementary RNAs and direct ribosome biogenesis (5-11). RNA-guided systems hold enormous potential for development of molecular tools in the laboratory thanks to their easy programmability. In the last decade, applications of bacterial CRISPR-Cas systems to genome and epigenome editing, as well as RNA editing, regulation and detection, have revolutionized basic bioscience, molecular medicine, and biotechnology. In addition, eukaryotic RNA interference has also been utilized as a molecular tool for gene knockdown. The recent discovery of the prokaryotic OMEGA systems (12, 13), the apparent ancestors of the effector nucleases of type II and type V CRISPR systems (Cas9 and Cas12, respectively), and their eukaryotic homologs, Fanzors (14-16), has substantially expanded the known diversity of RNA-guided systems. In parallel, CRISPR-associated transposons (17-22) and proteases (23-27) have added distinct RNA-guided functionalities beyond RNA-guided endonuclease activity to the repertoire of natural and potentially applicable RNA-guided molecular machinery. These RNA-guided systems were identified based on their evolutionary relationship with CRISPR-Cas, but to directly expand the diversity of RNA-guided systems, more general database mining methodologies are needed.

By integrating structural mining, sequence profile mining, and clustering via community detection from protein embeddings, we discovered a diverse family of RNA-guided systems encoded mostly by bacteriophages, archaeal viruses, and parasitic bacteria. These Tandem Interspaced Guide RNA (TIGR) systems feature proteins containing Nop domains, the hallmarks of the eukaryotic box C/D family sno ribonucleoproteins (RNPs) (28, 29). In TIGR systems, the Nop domain proteins are encoded adjacent to distinct, long arrays encompassing dual-spacer guides. These spacers are embedded within either dual-repeat units or stem-loop structures, with each unit repeated multiple times. We show that TIGR arrays are transcribed and processed into small RNA guides (tigRNAs), which direct TIGR-associated (Tas) proteins to target both strands of DNA in a PAM independent manner. The single effector proteins associated with TIGR arrays display diverse architectures, including instances where the core Nop domain is fused to HNH or RuvC nucleases. This modularity and versatility of effector protein architectures suggest that TIGR systems perform diverse biological roles, from RNA-guided cleavage of the target DNA to regulatory functions. We describe the discovery, biochemical characterization, and structural analysis of TIGR systems, which together provide crucial insights into their unique mechanisms and functions. We reveal evolutionary connections between TIGR and IS110 family transposons and box C/D snoRNAs, characterize their targeting rules, and demonstrate their potential as gene-editing tools, including RNA-guided DNA targeting in human cells.

## A family of RNAs associated with dual repeat arrays

**Figure 1.**
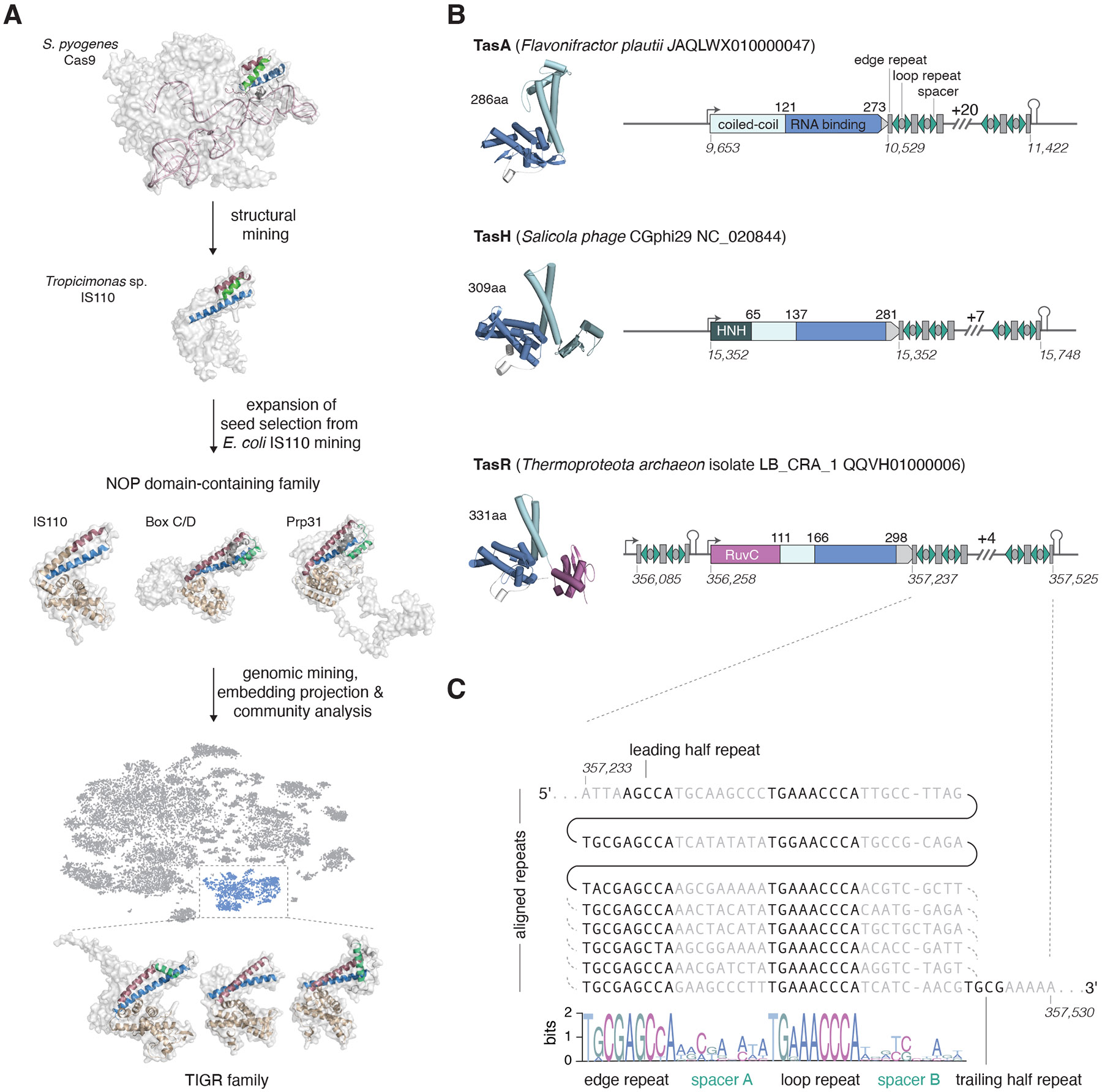
Discovery and Genomic Organization of TIGR Systems **(A)** Structural mining and identification of TIGR systems. The RNA-binding domain (RBD) of SpCas9 (PDB: 8G1I) was used as a seed to identify IS110 as a structural homolog. Further structural mining revealed similarity to the Nop domain-containing family. Genomic mining of Nop domain-containing proteins followed by community detection from embedding of the candidates identified a distinct family of Nop domain-containing proteins associated with Tandem Interspaced Guide RNA (TIGR) systems. Protein structures or models are all colored similarly: RBD, red, blue and green; rest of the Nop domain, wheat. **(B)** Genomic organization of TIGR systems and architectures of Tas proteins. TIGR-associated (Tas) protein structural models (left) and genomic locus architecture (right) of three representative Tas proteins (TasA, no nuclease; TasR, RuvC; TasH, HNH). Protein amino acid (top) and nucleotide (bottom) coordinates are shown, as are the number of repeat units in the TIGR arrays. **(C)** Sequence composition of TIGR arrays. Alignment of individual repeat units from the TaTIGR array downstream of the TasR ORF. Conserved regions corresponding to edge and loop repeats are shown in black, and variable regions corresponding to spacers A and B are shown in gray.

To identify previously unknown RNA-binding biological systems, we used the RNA-binding domain (RBD) of SpCas9, which interacts with the tracr::crRNA guide (Methods and Fig. 1A), as a query to search for structurally similar proteins in databases of experimental and modelled structures (Methods). Among the structural hits, we focused on a domain of the *Tropocimonas* IS110 transposon-encoded transposase, which showed structural similarity to SpCas9 RBD (DALI z-score 5.9) (Methods and fig. S1A). We confirmed experimentally that the IS110 transposase binds to a ncRNA required for transposition (Methods and fig. S2), and IS110 was recently demonstrated to function as a RNA-guided recombinase (30, 31). The region with structural similarity to SpCas9 interacts with the IS110 guide RNA, termed bridge RNA (30-32). This domain is found only in a small Cas9 clade that includes SpCas9 and its relatives (Methods and table S1) and its deletion impairs SpCas9 function in repression assays(33). It is possible that IS110 integrated into the *cas9* gene ancestral to this clade, and a part of the RBD was retained, likely contributing to guide RNA binding stabilization.

We then expanded this analysis by focusing on the RBD of IS110 itself. We selected the full RBD of *E. coli* IS110 as a seed for further exploration due to its smaller size and simpler architecture compared to other homologs, facilitating targeted analysis by being the least common denominator with potential structurally-related proteins (fig. S1B). Using this domain as the query to search protein structure databases (coupling Foldseek (34) and DALI (35, 36), see Methods), we identified structural similarity to the Nucleolar Protein (Nop) domain family (fig. S1C and 1D), a conserved RNA-binding domain superfamily shared by box C/D snoRNA systems (28) and the eukaryotic splicing factor Prp31 (29) (Fig. 1A and fig. S1C, 1D). The evolutionary link between IS110 transposases and the Nop domain has been recently reported independently (29, 37). These observations suggest that the RNA-binding capacity of the Nop domain can be used for diverse functions.

Prompted by these observations, we conducted large-scale mining for Nop domains across microbial genomic and metagenomic databases and coupled a community analysis-based clustering and dimensionality reduction from protein embeddings to define Nop domain-containing protein families (fig. S1E, 1F, Methods, and Fig. 1A). This approach uncovered a variety of distinct Nop domain families (19 Leiden communities), including IS110, box C/D snoRNPs, and Prp31. One large isolated family consisted primarily of proteins containing the Nop domain alone (Fig. 1A, fig. S1E), although subsets of these proteins encompassed additional N-terminal nuclease domains, including predicted active HNH and RuvC nucleases, or a predicted catalytically inactive RuvC domain (Fig. 1B and fig. S3).

Genomic context analysis showed that genes encoding Nop domain proteins of this family are associated with long arrays of interspaced repeats (Fig. 1B and 1C, fig. S4, Methods). Although reminiscent of CRISPR arrays, which typically consist of short (25–36 nt) repeats separated by variable spacers of a similar size (38), these arrays substantially differ in that they consist of two distinct, alternating 8–12-nt repeats, interspaced by 9-nt spacers (although there are some exceptions with spacers up to 12 nt) (Fig. 1C and fig. S4 and S5A). We name these arrays Tandem Interspaced Guide RNA (TIGR) arrays, and refer to their units as, sequentially: edge repeat, spacer A, loop repeat, and spacer B. A similar array structure was previously observed in an archaeal virus genome; however, its functional role was not investigated (39). The TIGR units are repeated multiple times (most often, 5 to 15) and are located upstream and/or downstream of the genes encoding the associated Nop proteins (Fig. 1B and fig. S4). TIGR arrays typically begin with a 5′ truncated edge repeat and end with a 3′ truncated edge repeat followed by a predicted rho-independent terminator (40) (Fig. 1C). We also noticed that the sequences of edge and loop repeats, as well as the spacer sizes, are conserved within a given locus but vary across different loci (fig. S4 and S5A).

We refer to these Nop domain–containing proteins as TIGR-associated (Tas) proteins: TasA, which is a solo Nop domain (e.g., in the *Flavonifractor plautii* locus, FpTIGR); TasH, which contains an HNH nuclease in its N-terminal region (e.g., in the *Salicola phage CGphi29* locus, SpTIGR); and TasR, which features a RuvC nuclease in its N-terminal region which is predicted to be either active (e.g., in the *Thermoproteota archaeon isolate LB_CRA_1* locus, TaTIGR) or inactivated (e.g., in the *Candidatus Buchananbacteria bacterium* RIFCSPHIGHO2_02_FULL_56_16 rifcsphigho2_02_scaffold_15087) (fig. S3D, Fig. 1B). Numerous TIGR loci were found in genomes of diverse tailed bacteriophages (41) and archaeal viruses (39) or in integrated proviruses, whereas others mapped to a variety of bacterial and archaeal genomes, in particular, those of parasitic bacteria of the Candidate Phyla Radiation (42) (CPR, or Patescibacteria (43)). For the majority of the identified TIGR loci, taxonomy assignment was difficult because they were located on metagenomic contigs (table S1).

## TIGR arrays are expressed and processed into 36-nucleotide RNAs

**Figure 2.**
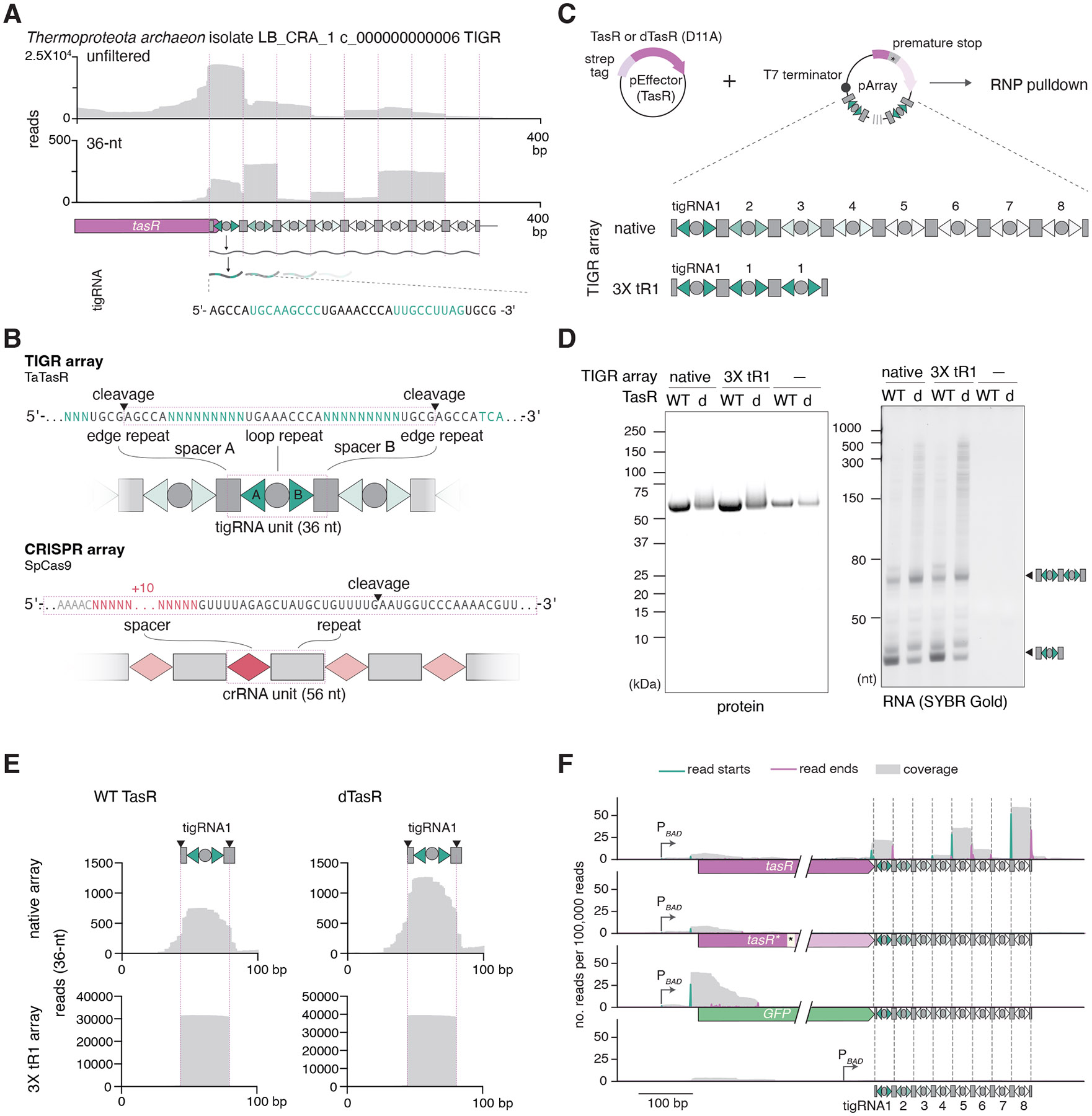
TIGR arrays are processed into 36-nt tigRNAs **(A)** Small RNA-seq of RNA pulled down with *Thermoproteota archaeon* TasR (TaTasR) mapped to native TaTIGR array expressed in *E. coli*. Top: All reads mapped to the TIGR array. Bottom: filtered 36-nt long reads. The pre-tigRNA transcript is expressed and processed into distinct 36-nt tigRNA units (see also fig. S5). **(B)** Top: Schematic representation of pre-tigRNA processing into tigRNA units. Bottom: Schematic representation of pre-crRNA processing (SpCas9 CRISPR system) into crRNA units. **(C)** Experimental design to test wild-type (WT) or catalytically-inactive TasR (dTasR, D11A) association with native tigRNA arrays or a minimal tigRNA array consisting of three repeats of tigRNA1 (3X tR1) via ribonucleic acid protein (RNP) pulldown. **(D)** Composition of purified TaTasR RNPs using the experimental design shown in (C). Left: SDS-PAGE protein gel stained with Coomassie blue. Right: 10% denaturing PAGE gel stained with SYBR Gold to show nucleic acids. **(E)** Small RNA-seq of RNAs present in TaTasR RNPs showing comparison between the processing of the native array (top) and the 3X tR1 array (bottom). **(F)** Small RNA-seq of total RNA from *E. coli*. The TIGR-TasR expression plasmid contained an arabinose-inducible promoter (P*BAD*) before the TasR gene, and cells were grown in the presence of arabinose. Coverage and read starts/ends are shown for reads that mapped to the expression plasmid. From top to bottom, the plasmid expresses the (1) WT system, (2) WT system with a nonsense mutation at Asp200 of TasR, (3) Residues 1-322 of TasR (331 residues total) replaced with GFP, and (4) Residues 1-322 to TasR deleted.

We hypothesized that TIGR arrays are transcribed into RNAs that would bind the respective Tas proteins. To test this hypothesis, we heterologously expressed Twin-Strep-SUMO tagged FpTasA, TaTasR, and SpTasH proteins with their respective downstream TIGR arrays in *E. coli* and performed pull-down experiments followed by small RNA sequencing (Fig. 2A and fig. S5B and C). We found that TasA, TasR, and TasH RNP complexes all contained 36-nt RNA species that started from the middle of an edge repeat, followed by spacer A, loop repeat, and spacer B, and ended in the middle of a second edge repeat (Fig. 2B). This processing pattern is reminiscent of CRISPR-Cas systems, where pre-crRNAs are processed to yield individual guide RNAs (44) (Fig. 2B) The distribution of read lengths obtained from these RNA-seq experiments typically showed a peak at 36 nt, along with peaks at its multiples, such as 72 nt and 108 nt, suggesting that a longer precursor RNA transcribed from a TIGR array is processed into multiple 36-nt RNA species which we denoted tigRNA (fig. S5D). The processing sites of tigRNAs appear to be precise, almost always occurring at the same position within an edge repeat. Minimized arrays containing one to three tigRNA units could be processed into TasR or TasH-binding tigRNAs, provided they had full edge repeats (Fig. 2C, D and fig. S6). We found that the nuclease activity of TasR was not required for processing (Fig. 2C-E), which is consistent with the observation that the TasA protein, which lacks any accessory domains or clear associations with other proteins (Fig. 1B), also binds to 36-nt RNA species (fig. S5B, 5D). However, the TasR protein itself appears to be required for RNA processing because a nonsense mutation, deletion of the TasR coding region, or replacement of TasR with GFP all resulted in loss of any detectable 36-nt RNA species after total small RNA sequencing (Fig. 2F). Collectively, these results indicate that Tas proteins bind to 36-nt tigRNAs generated from the TIGR array.

## Targeting rules of TIGR systems

**Figure 3.**
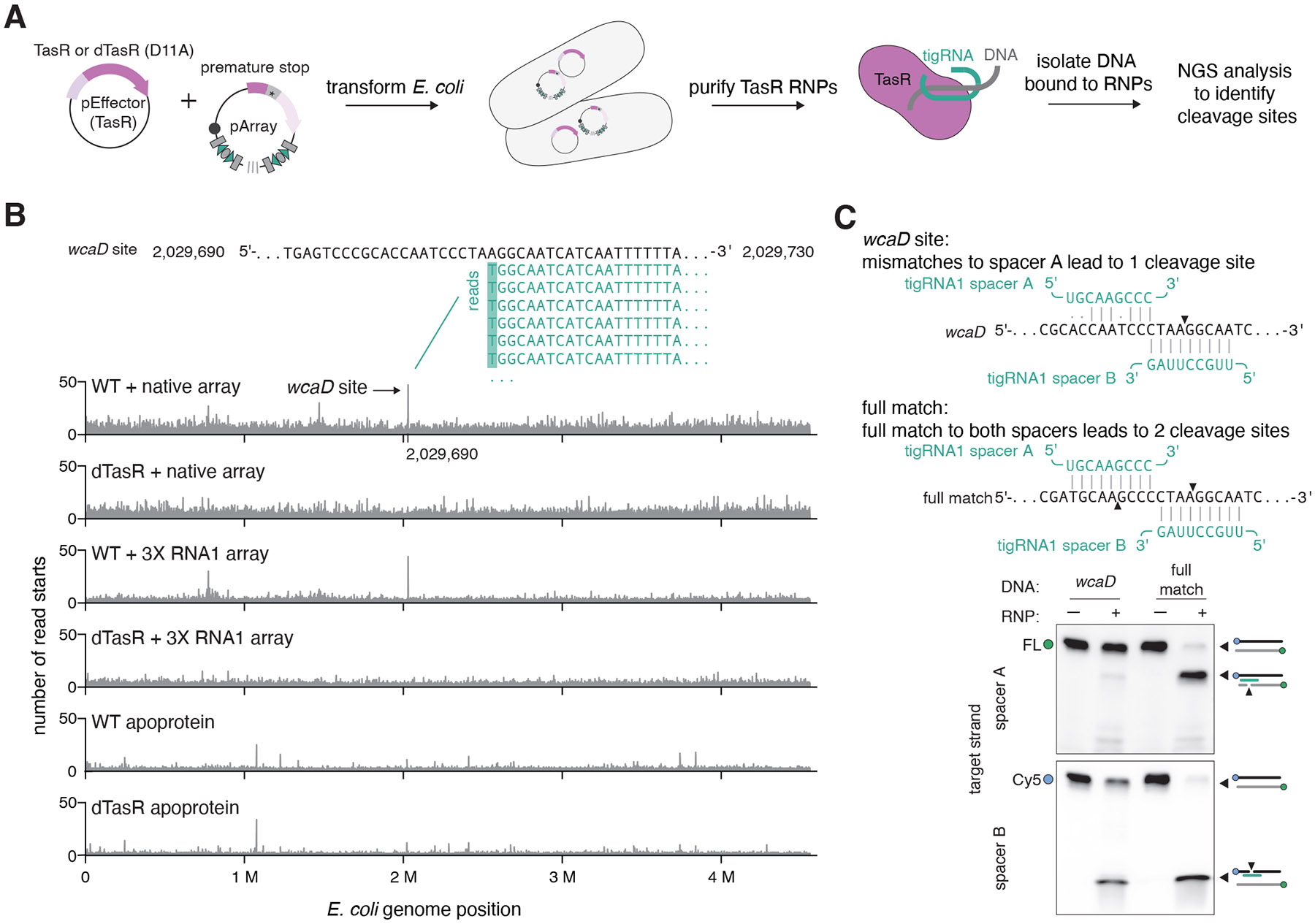
Identification of the TasR nuclease target **(A)** Experimental scheme to identify TasR cleavage sites in the *E. coli* genome. **(B)** Mapping of read starts to the *E. coli* genome. The annotated peak is at the *wcaD* gene. Example reads start with a ‘T’ due to the non-templated A added during NGS library preparation with Klenow DNA polymerase and identifies the 5′ end of the input DNA fragment. The pArray variant was either native, minimized to three identical units (3X tR1), or absent (apoprotein). **(C)** Top: The *wcaD* gene sequence with potential matches to spacer A and spacer B indicated, and the sequence of a full matching synthetic target. Bottom: denaturing polyacrylamide gel of an in vitro cleavage reaction using purified TasR RNP (coexpressed with a 3X tR1 TIGR array) and strand-specifically labelled substrates. FL, fluorescein label on the strand targeted by spacer A.

We hypothesized that tigRNA might function as a guide RNA for the Tas protein because each tigRNA harbors variable split spacers which combined represent a total of 18-nt variable sequence for FpTIGR and TaTIGR, and 16-nt for SpTIGR (fig. S5A). Given that SpTasH and TaTasR encompass nucleases in their N-terminal regions, we proposed that tigRNAs guide the Tas proteins to specific cleavage sites. How the split spacers would recognize a DNA target was not immediately obvious. During purification of TaTasR RNP from *E. coli*, we fortuitously noticed that this protein copurified with DNA. After sequencing the 5′ ends of the copurified DNA in a strand-specific manner, we found a prominent peak within the *E. coli wcaD* gene sequence (Fig. 3A, B). This peak was only present when TasR was coexpressed with the corresponding TIGR array and not when the TasR RuvC active site was mutated (Asp11 to alanine (D11A)), suggesting that the observed specificity depended on both tigRNA and the nuclease activity of the RuvC nuclease domain of TasR (Fig. 3B). We also repeated the experiment with a reprogrammed TIGR array (3X tR1) that only contained three copies of the first tigRNA (tigRNA1) of the array (which is still processed effectively, see Fig. 2D), and found that the peak in the *wcaD* gene was still present, suggesting that the targeting specificity depends on tigRNA1 (Fig. 3B). When we examined the peak, we found that the cleaved strand was fully complementary to spacer B of tigRNA1 whereas the adjacent region of the uncleaved strand could only form 6 out of 9 base pairs with spacer A (Fig. 3C).

**Figure 4∣:**
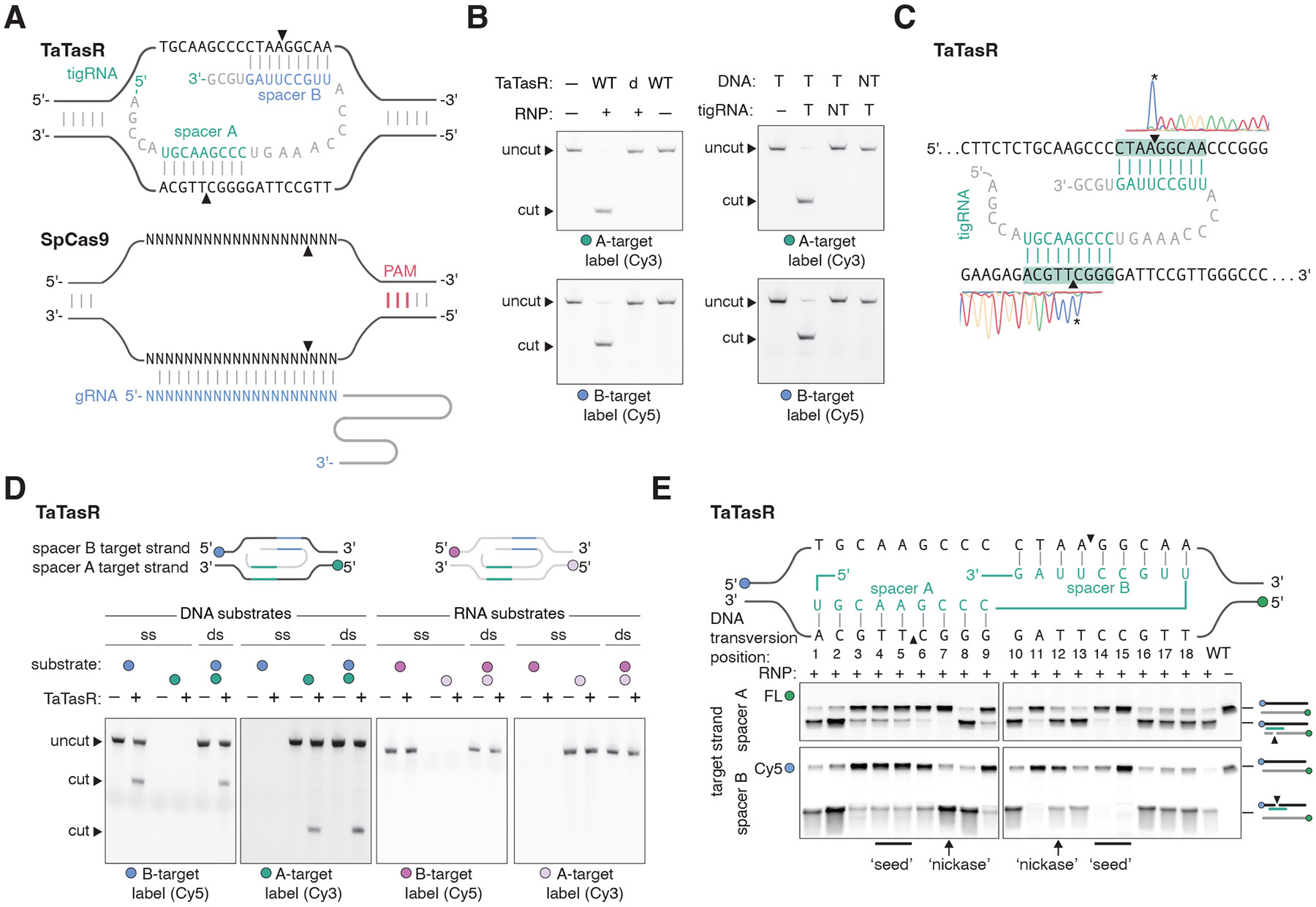
DNA targeting rules for TIGR-TasR **(A)** Top: base-pairing scheme of tigRNA1 from the TaTasR TIGR array with the synthetic optimized target sequence. Triangles indicate cleavage sites. Bottom: comparison with the targeting rules for CRISPR Cas9. PAM, protospacer-adjacent motif. **(B)** In vitro cleavage reactions with TasR. Left: TaTasR (WT) or catalytically inactivated RuvC domain (d, D11A mutation) RNPs were purified and incubated with synthetic target DNA matching the first tigRNA from the TaTasR TIGR array (RNP “–” indicates apoprotein was used). Right: Purified WT TaTasR apoprotein was incubated with the synthesized cognate tigRNA1, no RNA, or a non-targeting (NT) tigRNA, with the target (T) DNA substrate or non-target (NT) substrate. tigRNA2 from the TaTasR TIGR array was used as the non-targeting tigRNA, and PCR amplicon of the SpTasH target was used for the non-target DNA substrate. **(C)** Sanger traces for sequencing of the TasR in vitro-cleaved optimized DNA target. The polymerase used in Sanger sequencing adds a non-templated A after running off the template, indicated with an asterisk, and this delineates the precise cleavage site. **(D)** In vitro activity of TasR containing tigRNA1 on labelled single-stranded or double-stranded DNA or RNA substrates containing matches to spacer A and/or spacer B. **(E)** Effects of transversions on the in vitro cleavage of the optimized DNA target by TasR RNPs containing tigRNA1. All transversions were strictly A→T, T→A, G→C and C→G. Both strands of the target were mutated. The seed and nickase mutations are indicated. For (**B**, **D**, and **E**) reactions were resolved by denaturing PAGE and visualized by fluorescent labels on the DNA 5′ ends.

To test whether the sequence within the *wcaD* gene is a true target for TasR nuclease activity, we reconstituted cation-dependent TasR-directed DNA cleavage in vitro (Fig. 3C, fig. S7A). We prepared double-stranded DNA substrates in which each strand carried a different fluorescent label and incubated these with a TasR RNP containing copurified tigRNA1. We found that the strand complementary to spacer B was cleaved with moderate efficiency, whereas the strand with partial spacer A complementarity was mostly uncleaved, consistent with our sequencing results (Fig. 3C). When this strand was made fully complementary to tigRNA1 spacer A, both strands of the target were cleaved efficiently (Fig. 3C). Combined, our observations suggest that the spacers of tigRNAs specify the targets for Tas proteins by acting in tandem, with spacer A pairing to one strand of the target and spacer B pairing to the other strand (Fig. 4A). This RNA-guided mechanism is markedly distinct from the targeting mode of CRISPR systems where the crRNA spacer is fully complementary to a single strand of the target site (Fig. 4A).

## TasR and TasH are RNA-guided endonucleases

To test and further refine this model, we first showed that TasR nuclease activity against the optimized DNA substrate was dependent on the TasR RuvC active site (Fig. 4B). We observed no DNA targeting in the absence of a tigRNA, nor when the tigRNA spacers were mutated (Fig. 4B). To determine the precise sites of DNA cleavage by TasR, we used Sanger sequencing and found that each spacer specified the nicking site in an identical way, with each strand cut 3′ to the nucleotide complementary to the 5th base of the spacer, resulting in a double-strand break with 8-nt 3′ overhangs (Fig. 4C). TasR had no activity on single-stranded or double-stranded RNA substrates with spacer A and/or B matches but was able to precisely cleave single-stranded DNA containing matches to spacer A or B (Fig. 4D). To explore the tolerance for mismatches to tigRNA spacers, we made transversion mutations (i.e., T to A, G to C, etc) at each position of the DNA target and measured cleavage activity on each strand (Fig. 4E). We found that mismatches in the two nucleotides 3′ to the cut site, on either spacer matching sequence, were deleterious to cleavage of both strands. We refer to these positions as the ‘seed’ region, by analogy to the regions of essential base-pairing in microRNAs and CRISPR systems. Cleavage was more tolerant to mutations in positions closer to the 5′ or 3′ edges of the spacer-matching sequences, but double mutations at the 5′ edges of spacer-matching sequences impaired activity (Fig. 4E, fig. S7C). In general, we found that any mutation that impaired cleavage of one strand invariably also impaired cleavage of the other strand, suggesting the importance of both spacers working in tandem to identify a target site. This rule had one notable exception: a transversion introduced two nucleotides 5′ to the cut site only impaired cleavage of the strand for which the spacer pairing was impaired. Such mutations could be used to convert TasR into a nickase, and we refer to them as ‘nickase mutations’. Spacer A and spacer B sequences do not show obvious covariation, and thus are unlikely to overlap to specify target sites. However, we wondered whether gaps between the spacer A- and B-complementary sequences would be tolerated. We tested this, and found that 1- and 2-bp gaps allowed cleavage with efficiencies similar to that of the non-gapped target sequence, but any larger gap precluded cleavage (fig. S7D).

We also investigated the activity of tigRNA variants. tigRNAs only containing spacer A or spacer B sequences did not guide DNA cleavage, nor did tigRNAs with reverse-complemented spacer sequences. Notably, tigRNAs with swapped spacer A and spacer B sequences were functional for DNA cleavage, consistent with these having equivalent, symmetric roles in specifying DNA targets (fig. S7E).

Given that RNA-guided endonucleases belonging to CRISPR and OMEGA systems require a protospacer-adjacent motif (PAM) or target-adjacent motif (TAM) for efficient target DNA cleavage (12), we tested if Tas nucleases have a similar requirement. We adopted a previously developed TAM identification assay to sequence 8-bp randomized regions 5′ or 3′ to the TasR target sequence in a manner dependent on TasR-directed cleavage (fig. S7F, G). We found no strong enrichment of specific 5′ and 3′ sequences, indicating that the tigRNA-guided DNA cleavage by TasR only relies on target sequence information.

We extended our findings to a different TIGR nuclease, TasH, that contains an N-terminal HNH nuclease domain and a tigRNA guide with different edge and loop repeats, and found that TasH similarly cleaved the target DNA in an HNH- and tigRNA-dependent fashion when the opposite strands of the DNA target paired with the two tigRNA spacers, and that cleavage occurred in the same spacer position as in the case of TasR (fig. S8). Like TasR, we did not observe a targeting motif requirement for TasH (fig. S7G).

To test if the cleavage activity of TasR would be sufficient to restrict mobility of MGEs like a CRISPR system, we overexpressed TasR with a 3X tR1 array in recipient *E. coli* cells and transformed a target-containing plasmid by conjugation but found no reduction in conjugation efficiency compared to an untargeted control plasmid (fig. S9A). We also tried making a TIGR array with spacer pairs matching a phage genome but found no reduction in efficiency of plaquing (fig. S9B). Therefore, in this heterologous context, TasR nuclease activity does not appear to be strong enough to restrict MGE mobility. However, we did observe a strong effect of TasR on plasmid maintenance. A plasmid containing the tigRNA1 target sequence was lost from 99% of cells after overnight growth without antibiotic selection, whereas an untargeted plasmid was almost fully maintained, and plasmid loss was dependent on the TasR RuvC active site and the tigRNA sequence (fig. S9C, D). After daily passaging for four days without selection, 1 in 10^5^ cells maintained the targeted plasmid, and when we sequenced 188 of such cases, we found that 95% had acquired escape mutations in the spacer A or spacer B matching sequences including at the seed regions, and the remaining 5% had developed IS1 transposon insertions in the *tasR* promoter or TIGR array, supporting the conclusion that plasmid loss can be driven by TIGR-TasR activity (fig. S9E). Thus, although TasR could not inhibit uptake of MGEs in *E. coli*, it could drive their loss, suggesting its biological role could be in multigenerational MGE inheritance rather than defense.

**Figure 5∣:**
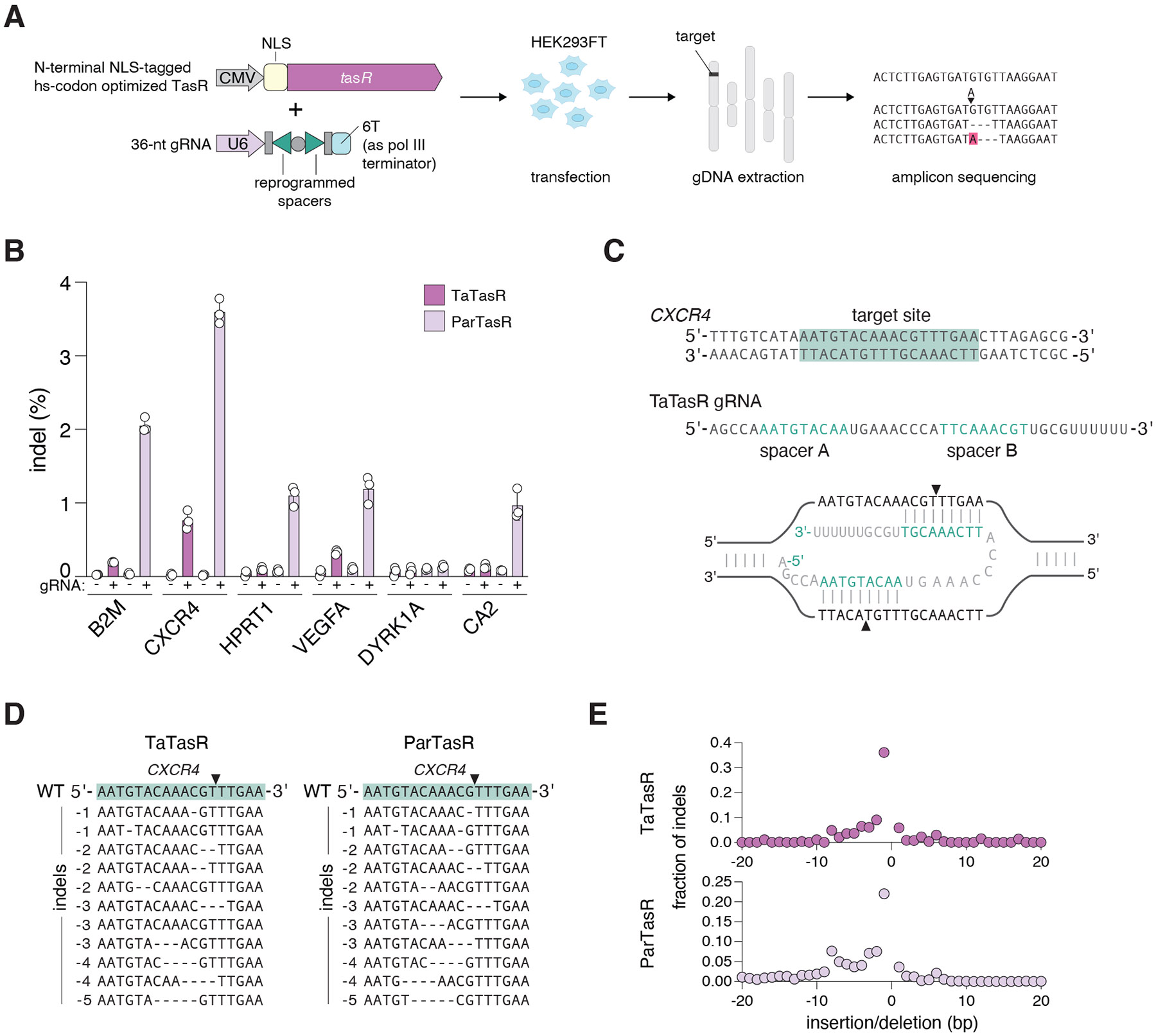
Human genome editing with TIGR-TasR **(A)** Experimental scheme to test programmable gene editing with TasR in HEK293FT cells. hs, Homo Sapiens; CMV, cytomegalovirus; NLS, nuclear localization signal. **(B)** Average indel rates (%) generated by TaTasR and a second TasR ortholog from Parcubacteria of the candidate phyla radiation (ParTasR) at six genomic loci in HEK293FT; data are presented as mean ± s.d. (n = 3). **(C)** Sequence of the *CXCR4* target site (highlighted in green) in the human genome and corresponding TasR gRNA with spacer matches shown in green. **(D)** Indels generated by TaTasR (left) and ParTasR (right) at the *CXCR4* target site. **(E)** Distribution of indel size generated by TaTasR (top) and ParTasR (bottom) at the *CXCR4* target site.

Based on the targeting and cleavage properties we observed, we then sought to adapt Tas nucleases for RNA-guided DNA cleavage in the genome of human cells. A plasmid encoding the human-codon-optimized Tas nuclease, either TaTasR or another TasR ortholog, from *Parcubacteria* of the candidate phyla radiation (ParTasR), tagged with a nuclear-localization signal (NLS) and the U6 promoter-driven 36-nt tigRNA expression plasmid were transiently transfected into HEK293FT cells. After 72 hours, genomic DNA was extracted and analyzed by amplicon sequencing for the presence of insertions and deletions (indels) at the targeted sites (Fig. 5A). To assess the programmability of the system, we screened six guides targeting six different loci: *B2M*, *CXCR4*, *HPRT1*, *VEGFA*, *DYRK1A*, and *CA2*. By screening the human genome editing activity of TasR orthologs in this experimental set-up, we confirmed that TaTasR and ParTasR both induced indels at these sites with varying efficiency (Fig. 5B). We observed the highest editing efficiency with both orthologs at the *CXCR4* locus: up to 0.8% indels with TaTasR and 3.6% with ParTasR (Fig. 5B, C). Deep sequencing of the target loci revealed that these two TasR nucleases had a similar smaller indel pattern, which is closer to the pattern observed for SpCas9 than those of ISDra2 TnpB, AsCas12a, and SpuFz1 (Fig. 5C-E). Overall, these data demonstrate the potential of TasR for human genome engineering applications. The lack of target motif requirement as well as the unique R-loop structure with two distinct ssDNA regions has the potential for developing base editors (45) and prime editors (46) with more flexible targeting and fewer bystander edits.

## Structure of a TasR RNP in complex with target DNA

**Figure 6∣:**
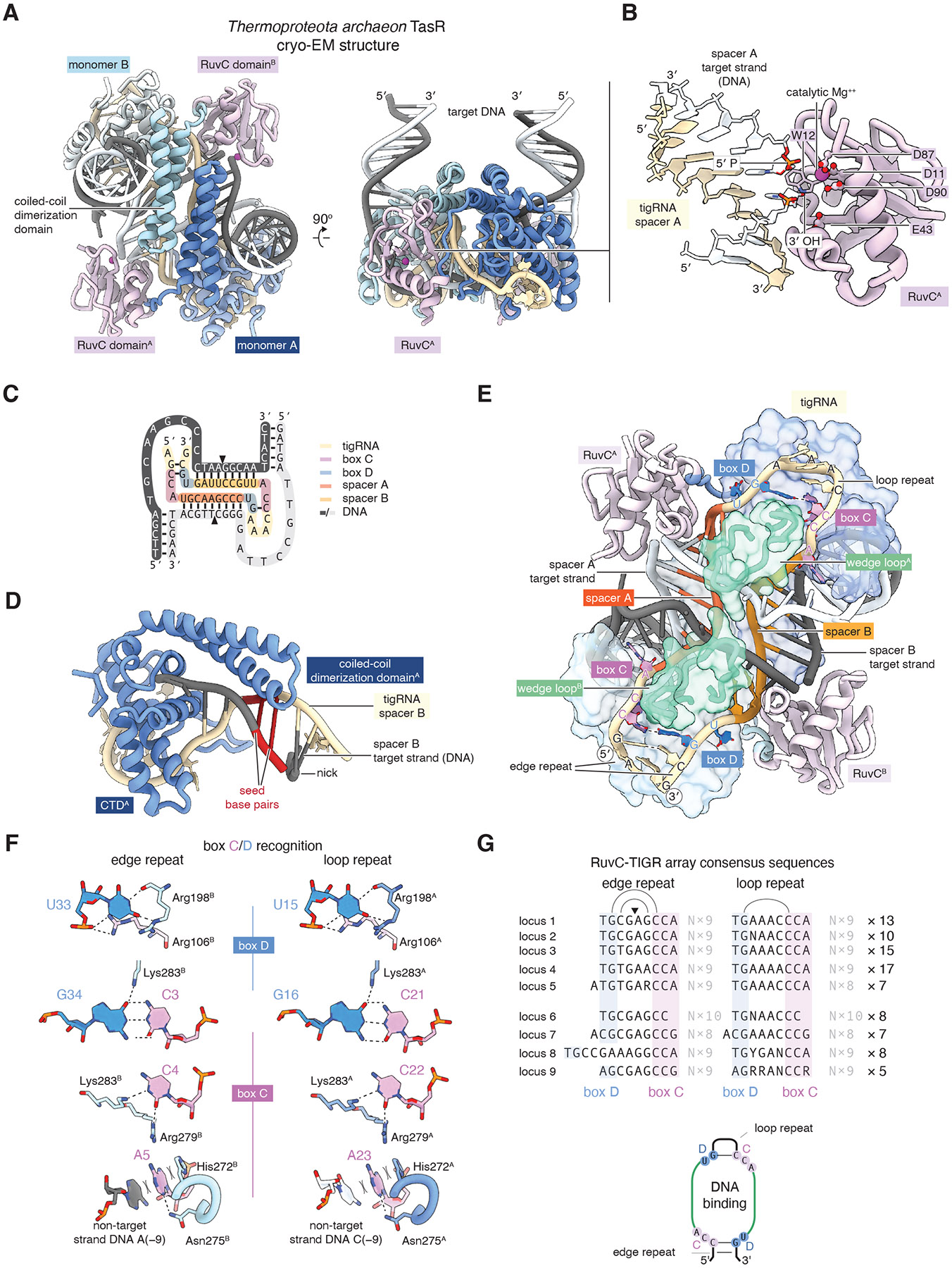
Structural basis for RNA-guided DNA cleavage by TIGR-TasR **(A)** Cryo-EM structure of TasR containing tigRNA1 and the optimized DNA substrate in a post-reaction product state. The protein is a dimer, and each protomer is labelled ‘A’ or ‘B’ according to whether its RuvC domain has cleaved the DNA strand targeted by spacer A or B. (**B**) Detail of the RuvC domain of monomer A interacting with the spacer A RNA/DNA heteroduplex. The 5′ phosphate of the nick is still in proximity to the RuvC active site, key residues of which are shown and labelled. (**C**) Diagram of the secondary structures of the RNA and DNA components of the TasR product complex. (**D**) Interaction of the coiled-coil dimerization domain of TasR monomer A with the seed region of the spacer B RNA/DNA heteroduplex (see Fig. 4E). CTD, C-terminal domain. (**E**) Recognition of the pseudosymmetric tigRNA by the symmetric TasR protein dimer. (**F**) Interactions of the box C and box D motifs of the edge (left) and loop (right) repeats with TasR residues. (**G**) Consensus sequences for edge and loop repeats from nine different TasR TIGR arrays. Excerpts from the full alignments used to derive these consensuses can be found in fig. S4. Arcs show the Watson/Crick base pairs observed in the structure (which are between different edge repeats on a processed tigRNA but are shown here on the same edge repeat for simplicity) which are either absolutely conserved (box C/D pairing) or show covariance.

To gain structural insights into the mechanism of RNA-guided DNA cleavage by TasR, we coexpressed TasR with the 3X tR1 tigRNA array, purified the resulting RNP, mixed it with a 60-bp dsDNA substrate containing the 18-bp optimized target sequence in the presence of magnesium to allow full cleavage, and solved the structure of the resulting product complex by cryogenic electron microscopy (cryo-EM) to 3.1 Å resolution (fig. S10, S11, table S2). The structure shows that the TasR protein folds into a canonical Nop domain and forms a C2-symmetric dimer via a coiled-coil domain in a manner identical to Nop5 of the archaeal box C/D snoRNP (Fig. 6A, fig. S12). The TasR dimer binds one copy of the target DNA and one copy of the 36-nt tigRNA. The cryo-EM density shows clear discontinuity at the expected cleavage position on both target DNA strands, consistent with the cryo-EM reconstruction capturing a product state, and each TasR RuvC domain active site is positioned in close proximity to each nick, with four highly conserved acidic residues located near the catalytic magnesium ion (Fig. 6B). The DNA substrate undergoes a dramatic 180° turn as it enters and exits the complex (Fig. 6A). The 18-bp target sequence itself is unwound, whereas all flanking sequences are fully base-paired in canonical B-form helices that do not form any base-specific interactions with the TasR protein or tigRNA, consistent with the lack of a targeting motif (Fig. 6C). All 9 nt of the spacer A-complementary strand are paired with tigRNA spacer A, forming an A-form helix, whereas the non-complementary strand is single stranded and is separated from its complementary strand by a ‘wedge loop’ insertion domain of the TasR protein. Spacer B and the spacer-B complementary strand are recognized in an identical manner. The minor groove of the tigRNA/DNA duplex rests on the TasR coiled-coil dimerization domain, and notably, the two base pairs that we identified as the ‘seed’ region make the closest interactions with the coiled coil, suggesting that the coiled-coil domain could increase fidelity by probing for correct helix geometry at the seed positions (Fig. 6D).

The 36-nt tigRNA forms a pseudosymmetric figure-eight shape as it interacts with both TasR protomers and both DNA target strands (Fig. 6E). This configuration, combined with the perfect symmetry of the TasR dimer, gives the whole complex pseudo C2 symmetry, which we were able to account for during cryoEM data processing as indicated by the distinct density for purines vs pyrimidines in spacers A and B (fig. S11). Once accounting for the pseudosymmetry, we observed that the edge repeat interacts with the Nop domain of one TasR protomer, whereas the loop repeat makes almost identical interactions with the other protomer. Specifically, both edge repeat and loop repeats start with a ‘UG’ sequence and end with a ‘CCA’ sequence. These motifs form identical base-specific interactions with different TasR protomers, and additionally, the underlined bases form Watson-Crick pairs in both repeats (Fig. 6F). Consensus sequences from other TasR TIGR arrays show that the ‘UG’ and ‘CCA’ motifs are highly conserved (Fig. 6G), and we observed that in TIGR arrays in general, these motifs could diverge but would almost always covary between the edge and loop repeats, consistent with a requirement to bind a symmetric protein dimer (fig. S4). By direct analogy to the equivalent sequences within box C/D snoRNAs (see discussion), we refer to the CCA motif as ‘box C’ and the UG motif as ‘box D’. These motifs form structural anchors for the tigRNA spacers and can be used to define a “C − 5 rule” that explains the cleavage sites for TasR and TasH (Fig. 4C, fig. S8), where the target DNA is cleaved 5′ to the nucleotide that pairs with the 5th tigRNA base downstream of the C box, analogous to the D + 5 rule for box C/D snoRNPs (47). The edge repeats and loop repeats can fundamentally be distinguished by the sequence that separates boxes C and D. In the TasR loop repeat, this sequence is A rich and forms a tetraloop-like structure that makes no clear sequence-specific contacts to the TasR protein. The TasR edge repeat contains a G-C pair additional to the box C/D pair, which in other TasR TIGR arrays can be a G-U or A-U pair but is always supported by covariation. The most 5′ and 3′ bases of the tigRNA could not be clearly visualized, precluding further insight into their specific processing mechanism. Nevertheless, we speculate that the two base pairs formed by the edge repeat may be important for their recognition by the putative processing endonuclease which might explain why TasR protein is required for tigRNA maturation, as these two solitary pairs would be unlikely to form in the naked tigRNA.

## Association of TIGR systems with arrays that contain conserved stem loop structures

**Figure 7∣:**
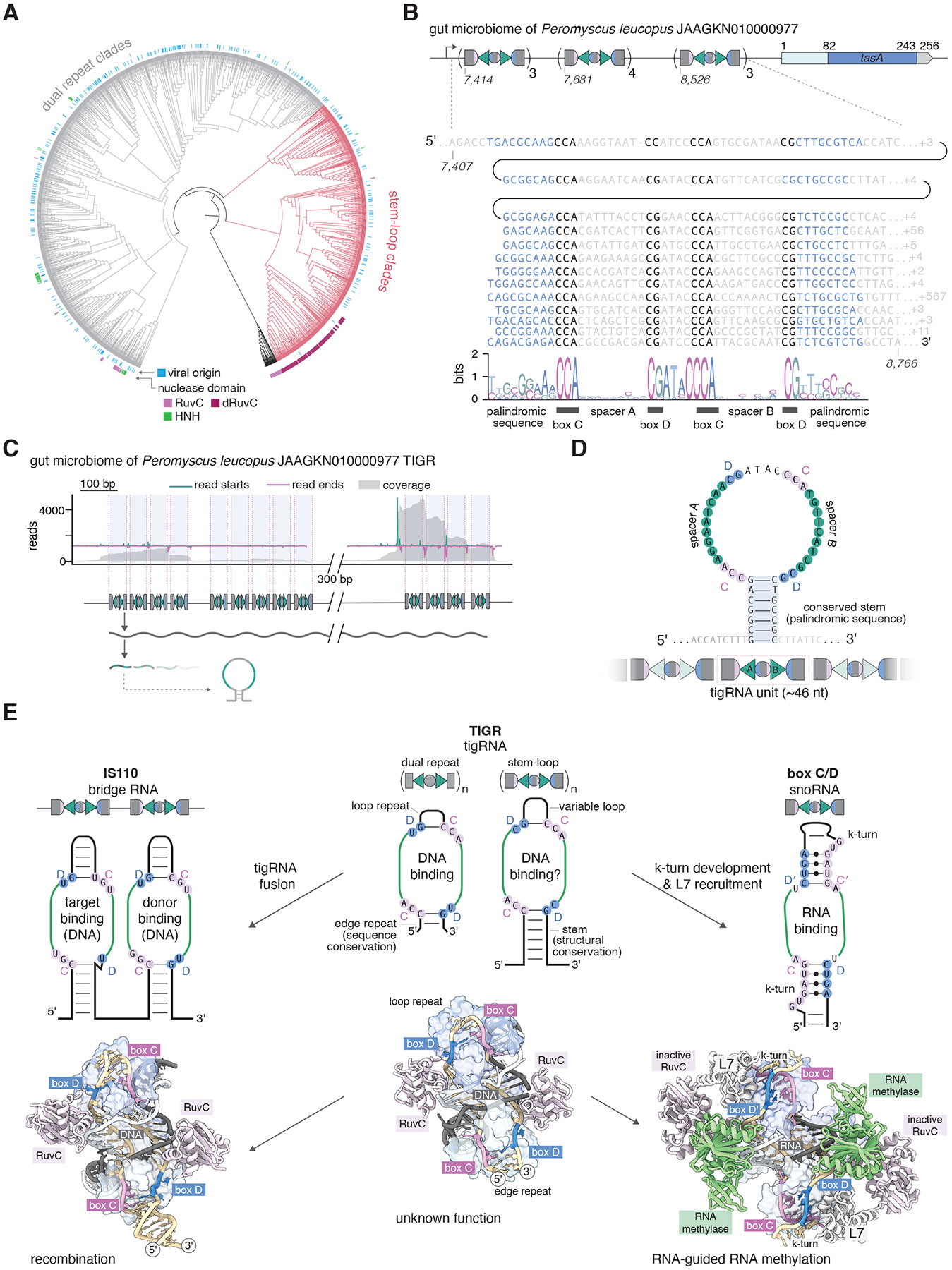
Evolutionary and functional diversity of TIGR systems. **(A)** Phylogenetic tree of the Tas Nop domain indicating array association, viral origin, and presence of RuvC (regardless of catalytic activity) or HNH nuclease domain. **(B)** Example of a TasA TIGR system with a stem-loop array (from the gut microbiome of *Peromyscus leucopus*). Top: locus architecture with TasA protein domain coordinates (in aa) above (coiled-coil region in light green; RNA binding C-terminal domain in blue) and nucleotide coordinates below. Bottom: Alignment of individual stem units, with hairpin sequences (palindromic sequence) in blue flanking each unit. Conserved C and D motifs are shown in black, spacers are in gray, and linkers between units are indicated. **(C)** Small RNA-seq mapping of the TIGR array following RNP pulldown. All three stem arrays are actively expressed and processed at linker regions. **(D)** Schematic of a representative stem-loop repeat unit (tigRNA2 from the TIGR system found in the gut microbiome of *Peromyscus leucopus*). **(E)** Comparative schematic of TIGR systems, IS110, and box C/D. tigRNAs are the simplest and shortest ncRNAs, while IS110 bridge RNAs resemble a fusion of two tigRNAs. box C/D snoRNAs are depicted as tigRNA-like molecules stabilizing the stem structure via the L7 k-turn motif. Bottom: comparison of the TasR-product complex with the crystal structure of an archaeal snoRNP (PDB 3PLA) (111) and a dimer excerpted from a cryo-EM structure of the IS110 bridge RNA complex (PDB 8WT6) (32). The RNP structures of all systems harbor the same domain architecture. TasR is structurally closer to IS110, while the box C/D protein (Nop5) harbors an inactive RuvC recruiting the fibrillarin methylase (green) and stabilizing the C and D box via an interaction with L7 (white). A proposed model positions TIGR as an ancestral system to IS110 and box C/D snoRNAs, highlighting its evolutionary relevance.

Tas proteins associated with dual repeat arrays form a distinct clade in the Nop domain phylogenetic tree, suggesting a common origin (Fig. 7A). In our exploration of the genomic neighborhoods of other Tas proteins outside this clade, we could not detect the dual repeat structure characteristic of TIGR arrays. Instead, in two clades we found a distinct locus architecture characterized by tandem stem-loops arrays (Fig. 7 and fig. S13A, B). Like the dual repeat TIGR arrays, these stem-loop arrays consist of two 8–12 nt spacers flanked by box C and box D motifs, but differ from dual repeats arrays in that they lack the separating repeats. Instead of edge repeats, these arrays contain variable but consistently palindromic sequences in the equivalent position that could form stem loop structures. Instead of loop repeats, these arrays contain variable intervening sequences that either show significant variation in size and sequence or maintain a conserved length and sequence, similar to true loop repeats in dual-repeat arrays. This observation suggests they may represent an intermediate stage between dual repeat arrays and stem loop arrays.

To experimentally characterize this stem loop-array architecture, we analyzed a stem-loop array from a contig sequenced from the gut microbiome of *Peromyscus leucopus* (table S1). This locus encodes a TasA protein lacking a nuclease domain and 13 stem-loop structures (Fig. 7B). The array is organized into three subarrays separated by a ~450-nt intergenic region. RNA-seq pull-down experiments confirmed that the array is expressed and processed into distinct modular units (Fig. 7C). Each processed RNA corresponds to a single stem-loop unit containing dual spacers flanked by the box C and box D motifs making the structure of the unit similar to snoRNAs. Plotting of RNA-seq read start and end positions indicated that the location of the cleavage is variable but does not affect the integrity of the hairpin (Fig. 7C and 7D). The existence of a second type of array architecture highlights the diversity of TIGR systems, while the conserved features shared between the two array types underscore the fundamental and minimal structure of the box C/D guide RNA (fig. S13). These findings suggest TIGR systems possess a high degree of modularity, positioning them as a promising foundation for the development of versatile genome-editing tools and paving the way for the discovery of additional RNA-guided systems rooted in box C/D-like architectures.

## Discussion

The discovery of Tandem Interspaced Guide RNA (TIGR) systems expands the known diversity of RNA-guided mechanisms. TIGR arrays exhibit a dual-spacer architecture with short spacers flanked by box C and box D motifs, within either dual-repeat units or stem-loop structures, and the spacers act in tandem to bind DNA targets, with each spacer targeting a different DNA strand. Why split a guide into two pieces? We speculate that this strategy allows TIGR systems to avoid targeting their own arrays, a role typically fulfilled by PAMs in CRISPR systems, but apparently absent in TIGR systems (fig. S7F, G). The compact size of individual spacers and repeats might also enhance TIGR array stability by minimizing recombination, a vulnerability exploited by phages integrating into CRISPR arrays (34, 48).

Many questions remain about the biology of TIGR systems. Most TIGR systems are prevalent in MGEs (Fig. 7A and table S1), including phages and archaeal viruses (39, 41), suggesting roles in defense against other MGEs, counter-defense against host immunity, gene regulation, or genetic mobility. Because TIGR systems are most prevalent in metagenomes, it is difficult to identify genuine targets for the spacers. We failed to observe any protection against plasmid conjugation or phage infection when expressing TasR in *E. coli*, but did observe strong inhibition of plasmid maintenance, suggesting a plausible role in biasing the inheritance of MGEs (fig. S9), however this heterologous expression might not reflect a native context. Whereas the Tas nucleases likely contribute to host-virus dynamics and/or inter-MGE competition by cleaving target DNA, the functions of nuclease-lacking TasA proteins are less apparent. These proteins might mediate inter-MGE competition via a mechanism that does not involve cleavage, such as blocking transcription or replication, as demonstrated for type IV (49-51) and type V-M CRISPR systems (52) that are typically encoded by plasmids (and less commonly, phages) and interfere with other MGEs without cleaving the DNA. The nearly 180° target DNA bend induced by TasR is likely also induced by TasA, and is reminiscent of the DNA bends introduced by lysogenic phage during recombination, suggesting TIGR-TasA systems could act as RNA-guided aids to phage integrases (53). Intriguingly, TIGR TasA loci are occasionally operonized with genes encoding proteins homologous to ParB, a DNA-binding protein involved in plasmid segregation (54) (fig. S14), suggesting additional functional diversity.

Another enigma is the mechanism of the emergence, maintenance, and expansion of TIGR arrays. CRISPR systems use a dedicated adaptation machinery containing Cas1 and Cas2 proteins to integrate MGE-derived DNA fragments into the leader-proximal end of the CRISPR arrays, resulting in the proximal repeat duplication (55); conversely, spacers can be lost from CRISPR arrays over time by recombination (56). TIGR systems generally consist of a single protein associated with the array and are not associated with any proteins that could be implicated in adaptation although it should be noted that some CRISPR systems lack adaptation genes and rely on those associated with other CRISPR loci (57). The dual-spacer organization poses additional challenges for array expansion mechanisms. Elucidating the origins of spacers in TIGR arrays will be critical to the identification of their natural targets and gaining insights into their biological roles.

The maturation mechanism of tigRNAs remains unknown as well. Our analysis shows that Tas proteins are required for processing but do not catalyze pre-tigRNA cleavage directly, even in those cases when they contain nuclease domains. Given that TIGR systems from diverse, phylogenetically distant organisms were consistently and precisely processed upon heterologous expression in *E. coli*, we suspect that a highly conserved host RNase is responsible for maturation. In type II and many type V CRISPR systems, pre-crRNAs are processed by the host RNase III (58-61) in conjunction with an accessory tracrRNA that forms a duplex with the pre-crRNA. Polycistronic snoRNAs in plants and some yeasts have also been shown to be processed by their respective RNase III homolog (62, 63). However, we were unable to identify any accessory RNA associated with TIGR arrays that could perform a similar role, and unprocessed TIGR arrays do not appear to form the extended dsRNA duplexes typically required for RNase III recognition. Thus, processing of the pre-tigRNA might likely rely on another highly conserved RNase that remains to be identified.

Despite these open questions about the biological role of TIGRs, our results highlight striking evolutionary connections between TIGR systems, IS110 transposons, and box C/D snoRNAs. All guide RNAs in these systems share a conserved tandem spacer architecture and are flanked by box C and box D motifs, and these box C/D motifs all bind to identical sites in the Nop domain (Fig. 7E). However, the flanking RNA structures are different: tigRNA loop and edge repeats are highly compact, whereas TIGR stem-loop repeat units likely form more extended RNA helices similar to those in IS110 bridge RNAs (Fig. 7E). By contrast, snoRNAs are flanked by K-turn motifs that recruit the L7 protein to form a more extensive RNA-protein interface (Fig. 7E). The organizational differences between these systems underscores their functional divergence. tigRNAs are clustered in highly regular arrays, whereas bridge RNAs in IS110 comprise a single two-stem unit encompassing target and donor information to perform recombination. By contrast, snoRNAs are more sparsely distributed across genomes, although clustered snoRNA arrays have been observed in archaea (64), plants (65-67), yeast, and vertebrates (65, 68-71).

The structural similarities between TIGR systems, IS110 transposons and box C/D snoRNPs allows us to propose a possible evolutionary link between these systems. It seems likely that the solo-Nop domain protein TasA, which is the effector most commonly found in TIGR systems, is the ancestral form to which nuclease domains were fused on multiple, independent occasions during evolution, yielding distinct functionalities (Fig. 7A). One fusion event involving a DEDD transposase, a protein sharing a common fold but not specifically related to the TasR RuvC domain, likely gave rise to IS110, which employs its bridge RNA for RNA-guided transposition and spread widely across the diversity of bacteria and archaea. The box C/D snoRNP Nop-domain protein also contains an N-terminal RuvC nuclease fusion, but this nuclease is inactive and instead recruits the fibrillarin methyltransferase as an effector instead. Interestingly, we identified a distinct clade of TasR proteins in CPR bacteria that is characterized by an inactive RuvC domain that interacts with large insertions in the Nop domain (table S1 and fig. S4) and is associated with stem arrays. This specialized clade of TasR proteins may represent a potential intermediate step toward the RNA-guided RNA modification systems of box C/D snoRNAs, though the precise evolutionary pathway and origins remain uncertain and warrant further investigation. On top of RNA-guided RNA methylation by box C/D snoRNPs, eukaryotes and archaea also employ a distinct family of H/ACA box snoRNPs to perform RNA-guided RNA pseudouridylation(7), and in the future it will be interesting to explore whether prokaryotes harbor RNA-guided systems related to H/ACA snoRNAs.

In conclusion, our study has revealed the existence of an extensive family of RNA-guided proteins in prokaryotes and their viruses. Their compact architecture, motif-free targeting, and tandem-spacer configuration make TIGR systems a promising platform for future biotechnology applications. Moreover, the structural and functional characterization of these systems reveals their evolutionary relationship to snoRNAs, providing a key missing link in understanding the emergence of RNA-guided targeting systems across life.

## Materials and Methods

### Structural similarity between SpCas9 RNA binding domain and IS110

To identify structural homologs of RNA-binding domains across Cas9 representatives, we performed a comprehensive structural search using DALI software on the AlphaFold database, clustered at 50% sequence identity using MMseqs2. Structural hits with a DALI score > 5 were retained and manually curated, with priority given to candidates based on functional annotations and predictions using HHpred (72). Among the hits, the *Tropicimonas sp. IMCC6043* IS110 (TroIS110) protein exhibited a notable similarity to the RNA-binding domain of *Erysipelotrichia bacterium* Cas9 (EbCas9), a representative structurally similar to SpCas9 (~30% of sequence identity) (as identified in (12); table S1). This domain interacts with the bulge and lower region of the tracrRNA::crRNA duplex in SpCas9 (PDB: 8G1I) (fig. S1A). The structural similarity between TroIS110 and EbCas9 was characterized by a DALI (35, 36) score of 5.9, alignment over 76 amino acids, and a root-mean-square deviation (RMSD) of 3.5 Å. Mapping the aligned regions onto the SpCas9 structure using PyMOL (The PyMOL Molecular Graphics System, Version 3.0 Schrödinger, LLC.) confirmed correspondence between SpCas9 positions 315–411 and TroIS110 positions 126–207 (fig. S1A), and we therefore performed a deeper investigation into IS110.

### *Bifidobacterium* IS110 is a RNP dependent transposase

RNA-guided activity of *Bifidobacterium* IS110 (BbIS110) was tested in a transposition assay (fig. S2). 100 ng of pHelper was co-electroporated with 100 ng of pR6K-donor and 100 ng of pTarget into One Shot^™^ PIR1 Chemically Competent *E. coli* (ThermoFischer) and plated on 50 mg/ml kanamycin, 50 mg/ml spectinomycin, and 50 mg/ml chloramphenicol containing LB-agar plates. After incubation for 17 hours at 37°C, all colonies were scraped, and plasmid DNA was purified using a QIAprep Spin Miniprep Kit (QIAGEN). The frequency of insertions was determined with droplet digital PCR (ddPCR) with 1 pg to 10 ng template plasmid DNA for 20 μL ddPCR reaction. Insertion events were quantified using insertion-specific primers and a donor-specific probe. ddPCR Supermix for Probes (No dUTP) (BioRad), primers (900 nM each), a probe (250 nM), and template DNA were combined into 20 μL reactions, and droplets were generated with 70 μL of Droplet Generation Oil for Probes (BioRad) using the QX200 Droplet Generator (BioRad). Thermal cycling conditions for ddPCR reactions were as follows: 1 cycle, 95°C, 10 min; 40 cycles, 94°C, 30 s, 58°C, 1 min; 1 cycle, 98°C, 10 mins; 4°C hold. PCR products were read with a QX200 Droplet Reader, and the absolute concentrations of inserts were determined using QuantaSoft (v1.6.6.0320). Total template (genome or target plasmid) amount was also quantified through this process, and insertion frequency was calculated as inserts/template. All data points are shown with an error bar showing standard deviation, and statistical significance was assessed by t-test.

### Mining of IS110 RNA binding domain

#### Selecting the seed.

We analyzed the C-terminal domain (CTD) of *E. coli* IS110 (EcIS110), excluding the RuvC domain, to investigate its structural and functional relationships. This region encompasses the domain that is structurally similar to the RNA-binding domain of SpCas9. Unlike the TroIS110 counterpart, EcIS110 features a shorter linker connecting the first two helices within the coiled-coil domain. Additionally, we extended this seed into the adjacent C-terminal segment, which is known to interact with the guide RNA and DNA duplex in IS110 (32).

#### Structural mining.

We first performed a structural similarity search in the following databases (fig. S1B):

- AlphaFold UniProt clustered at 50% sequence identity using mmseqs2 (73-75)
- MGnify structural database folded with Esmfold and clustered at 50% sequence identity (75, 76).
- BFVD, a viral database containing alphafold (73, 74) predicted structure of viral representatives of UniRef30 sequences (77).

A combination of Foldseek (34) and DALI (35, 36) was used in the following workflow:

- Step 1: Foldseek was used to identify candidate hits above a threshold of 20 bit-score, resulting in 28,902 hits.
- Step 2: DALI was employed to perform structural alignments of all hits against the IS110 CTD seed structure, selecting candidates with a Z-score > 6.0. This filtering step yielded 23,937 final candidates.
- Step 3: Pairwise alignments using DALI allowed masking of positions aligned with the seed structure while discarding gapped positions.

#### Phylogenetic Tree Construction.

Masked alignments were concatenated into a multiple sequence alignment, from which a phylogenetic tree was constructed using FastTree (78) with the Whelan and Goldman (-wag) substitution model (79). The resulting tree was visualized using iTOL (80, 81), with annotations indicating the presence or absence of the RuvC domain based on structural similarity with the EcIS110 RuvC domain. One clade contains Prp31 (no RuvC) and box C/D snoRNP Nop proteins (dead RuvC-like) suggesting a structural relationship between the RNA binding domain of IS110 and the Nop domain family found in the U4 snRNP and box C/D snoRNPs (37). One distinct clade lacks RuvC and was used to build a custom HHM profile (data S1 and fig. S1C and 1D).

### Large-Scale Sequence Mining and Leiden Analysis

#### Profile mining.

12 conserved profiles associated with IS110, box C/D, and Prp31 protein families were identified: COG1498, COG3547, KOG2572, KOG2573, KOG2574, PF01548, PF01798, PF02371, PF08156, PF09785, PF21572, and PF23667. Additionally, a custom HMM profile derived from the branch of interest identified during structural mining was included (data S1). These profiles were used to search for homologs across a custom microbial database comprising JGI, MG-RAST, NCBI, WGS, and EMBL compiled in (31, 82). HMMER’s hmmsearch (83) (hmmer.org version 3.4) was used to screen these 13 HMM profiles. Hits with an HMMER score > 15 were retained, resulting in 3,951,425 hits.

#### Clustering of Sequences.

Sequences were clustered (fig. S1E) using MMseqs2 (75) with the following steps:

- Step 1: Clustering at 100% sequence identity and 30% coverage (cov-mode 1) to remove partial proteins, yielding 1,049,166 unique sequences.
- Step 2: Further clustering at 70% sequence identity and 70% coverage, resulting in 136,768 groups.
- Step 3: Final clustering at 50% sequence identity and 70% coverage (50/70), reducing the dataset to 47,428 representative sequences.

#### Structural Selection of Candidates.

The 47,428 representatives were folded using AlphaFold2 via local ColabFold with default parameters (fig. S1E). Structural alignments against an AlphaFold-generated IS110 Nop region model were performed using DALI. Candidates with a DALI score > 1 (n = 24,376) were retained for further analysis. To annotate the presence of RuvC, a second round of structural comparisons was conducted using the AlphaFold-generated RuvC domain of EcIS110 with DALI.

#### Community Detection and Dimensionality Reduction.

From the DALI pairwise alignment with the IS110 Nop domain, continuous protein segments aligned with the query were extracted, excluding regions outside the IS110 Nop domain mask (upstream or downstream) (fig. S1E). The mean embedding of the final layer (48th layer) of each segment was computed using the ESM2 15B model (76), generating a 5,120-dimensional vector for each protein segment. A cosine similarity matrix of the embeddings was generated and analyzed using the Leiden algorithm (84), an improved version of the Louvain algorithm (85). Partitioning was performed with a resolution parameter of 0.5 to prevent over-clustering, using the Reichardt and Bornholdt Potts model (86) with a configuration null model. To visualize the 19 detected communities, embeddings were projected into two dimensions using t-SNE (87). Initialization was performed using PCA with the top 50 components, followed by t-SNE with a perplexity of 30 and a learning rate of 1,000.

### Identification of a novel Nop domain-containing family

Several communities included IS110-like proteins containing the RuvC N-terminal domain and the Nop C-terminal domain, exhibiting distinct structural variations, particularly within the Nop domain. Other communities contained a mixture of full IS110-like protein and truncated IS110-like proteins, evidenced by the sporadic absence of the RuvC domain. One community lacked clear structural similarity to the Nop domain, likely resulting from false-positive selection during clustering. An isolated community encompassed Prp31 and box C/D proteins, which were visually separated in the t-SNE (fig. S 1F). Adjacent to this group, we identified a large and dense cluster corresponding to Leiden community 2 (Fig. 1 and fig. S1F), in which the majority of proteins (82%) lacked the RuvC domain. This cluster corresponds to our candidates of interest identified through structural mining (from which our custom hmm profile was derived). The proximity of this group to box C/D and Prp31 groups suggests a potential relationship between them. We extracted the 2,200 representative members of this group of interest (table S1). These 2,200 candidates represent a total of 21,385 proteins (cluster members) that were analyzed further (table S1) and belong to a family we termed Tandem Interspaced Guide RNA (TIGR) systems encoding TIGR associated proteins (Tas). To better understand the evolutionary relationships within this group, we constructed a phylogenetic tree of Tas proteins using masked alignments as described above.

### Structural Organization of the Genomic Vicinity

#### Pattern Identification for TIGR Dual Repeat Arrays.

To assess the presence of non-coding RNAs, we analyzed the genomic context of the identified protein candidates. From the protein phylogenetic tree, we selected branches with strong bootstrap support (>0.9) and extracted 5 kb (or less for sequences near contig edges) of upstream and downstream nucleotide sequence. To identify conserved motifs, we employed MEME (88) (searching for motifs of 20–30 nt with a maximum of 1,000,000 motifs) and GLAM2 (88) (10 iterations). Results from MEME and GLAM2 were curated alongside multiple sequence alignments of 3–5 loci with similar protein sequences. Repeats and approximately 30 nt of flanking sequences were extracted, aligned using MAFFT (89), and manually adjusted to refine the alignment. Consensus sequences were derived from the repeats, and Geneious was used to locate motifs with 2–3 mismatches. This process was iterated to refine motif definitions. This approach allows us to identify several dual repeat TIGR arrays.

#### Pattern Identification for TIGR Stem Array.

For the branches lacking dual repeat arrays, we occasionally observed the presence of regular palindromic sequences surrounding the protein, suggesting the formation of hairpin-like structures. MEME (88) sometimes identified conserved 2–3 nucleotide motifs embedded within the loops of these hairpins. Using RNA2Drawer (90), local alignments of multiple hairpins, and MEME predictions, we identified two distinct conserved motifs (similarly positioned as the box C/D motifs) interspaced; this pattern is repeated twice within the hairpins’ loops with a variable linker in between (fig. S12).

### Taxonomy Prediction

The majority of contigs bearing TIGR systems originate from metagenomic sequences and lack species-level annotations. To assign taxonomy, we extracted the largest contig associated with each representative and used MetaBuli (91) to predict the taxonomic classification. For viral origin predictions, we extracted 10 kb of sequence both upstream and downstream of the gene of interest and ran ViralVerify (92) and PhageBoost (92, 93) to identify potential viral origins. Detailed results, including taxonomic and viral classifications, are provided in table S1.

### Genomic feature prediction

Rho-independent terminators were predicted using the ARNold webserver (40) (http://rssf.i2bc.paris-saclay.fr).

### Plasmid construction

Orthologs used in this study are listed in table S1. All plasmids used in this study are described in table S3. Plasmids were constructed using general cloning methodologies including Gibson assembly with NEBuilder HiFi DNA Assembly Master Mix (New England Biolabs, E2621L) and golden gate assembly (GGA) with a variety of type IIS restriction enzymes. The Stbl3 *E. coli* strain (Thermo Fisher, C737303) was used for DNA cloning. The sequences of cloned constructs were confirmed by whole-plasmid sequencing following Tn5 tagmentation after mini-prep of plasmids with QIAprep reagents (QIAGEN, 27106). For Tas protein expression in *E. coli*, gene fragments were cloned into a pET28 expression vector with a Twin-Strep-SUMO or Twin-Strep affinity tags. For the tigRNA array expression vectors, each TIGR system containing both the protein and the array was cloned into a pACYC expression vector. For the motif identification assay (fig. S7F, G), pTarget containing an 8N motif upstream of the protospacer 1 (PSP1) sequence was derived from pUC19 vector and the detailed information is described in previous studies. Human-codon optimized TasR was cloned into a pCMV expression vector with an N-terminal nuclear localization signal (NLS) and HA tag. The edge repeats of tigRNA with two inverted BbsI type IIS restriction sites were cloned behind the U6 promoter. Split guides with a loop repeat were cloned into the scaffolds by GGA as two annealed complementary oligonucleotides.

### Protein purification for copurified RNA and/or DNA sequencing

Tas protein expression vectors and/or tigRNA array expression vectors were transformed into electrocompetent BL21(DE3) Competent Cells (Sigma, CMC0016). Starter cultures (2 mL) were grown in TB supplemented with 50 μg/ml Kanamycin and/or 25 μg/ml Chloramphenicol for 12 h, which was used to inoculate 1 L of TB for growth at 37°C and 150 rpm until an OD600 of 0.6 was reached. Protein expression was induced by supplementation with IPTG to a final concentration of 0.5 mM. The cells were incubated at 16°C for 16 h for protein expression, and then harvested by centrifugation for 20 min at 4°C at 4000 *g* (Beckman Coulter Avanti J-E, rotor JLA8.100). All subsequent steps were performed at 4°C. Cell pellet was resuspended in 50 mL of lysis buffer (50 mM Tris-HCl, 500 mM NaCl, 1 mM DTT, pH 8.0) supplemented with cOmplete Protease Inhibitor Cocktail (Millipore sigma 4693116001). Cells were disrupted by the LM20 Microfluidizer system at 28,000 PSI. Lysate was cleared by centrifugation for 30 min at 4°C at 15000 *g* (Beckman Coulter Avanti J-E, rotor JLA-10.500). The cleared lysate was applied to 1 mL of packed Strep-Tactin Sepharose resin (IBA, 2-1201-002) and incubated with rotation for 1 h, followed by washing of the protein-bound beads in lysis buffer. The beads were also washed in high salt buffer (25 mM Tris-HCl, 1 M NaCl, 1 mM DTT, pH 8.0) to remove contaminating nucleotides. The proteins were eluted in lysis buffer supplemented with 2.5 mM d-desthiobiotin (Sigma, D1411). Resulting proteins were concentrated by an Amicon Ultra Centrifugal Filter Units (Millipore), and protein concentration was estimated by NuPAGE (Invitrogen) and Coomassie Instant Blue (Abcam, ab119211). All purified proteins were snap frozen and stored at −80°C.

### Small RNA sequencing

To sequence small RNAs that copurified with Tas proteins, RNA was extracted from RNPs purified from *E. coli* using a Direct-zol RNA kit (Zymo). Extracted RNA was treated with 10 units of DNase I (NEB, M0303) for 30 minutes at 37°C to remove residual DNA and purified again with an RNA Clean & Concentrator-25 kit (Zymo). The 2 μg of purified DNA-free RNA was then treated with 20 units of T4 polynucleotide kinase (PNK; NEB, M0201) for 1 hour at 37°C, purified and treated with 20 units of 5’ RNA polyphosphatase (Lucigen, RP8092H) for 30 minutes at 37°C and purified again. 1 μg of modified RNA was used as input to an NEBNext Small RNA Library Prep for Illumina (NEB, E7330). Amplified libraries were gel extracted and sequenced on an Illumina MiSeq with Read1 125 cycles, Read2 125 cycles and Index1 6 cycles. Adapters were trimmed using CutAdapt and mapped to loci of interest using Bowtie2.

To sequence total small RNAs from *E. coli*, strain Stbl3 containing either plasmids pMW357, pMW376, pMW378, or pMW380 were grown overnight at 37°C in LB + ampicillin + 0.2% (w/v) arabinose. Cultures were then used to inoculate fresh LB + ampicillin + 0.2% (w/v) arabinose and were grown to OD600 of 0.9. Cells were collected by centrifugation (4000 *g* for 5 min) and RNA was extracted using a Direct-zol RNA kit (Zymo) with on-column treatment with 30 U of DNase I (NEB, M0303). 1.5 μg was depleted of ribosomal RNA using the RiboMinus bacteria module (Invitrogen), purified, and treated with 20 U T4 PNK (NEB, M0201) for 60 minutes at 37°C. The RNA was purified again and used as an input to the NEBNext Small RNA Library Prep for Illumina (NEB, E7330). Amplified libraries were gel extracted and sequenced on an Illumina MiSeq with Read1 150 cycles, Read2 150 cycles and Index1 8 cycles. Adapters were trimmed using CutAdapt and mapped to the *E. coli* genome and TIGR expression plasmid using bwa mem.

### Cleaved single-stranded DNA sequencing

TasR RNP, purified as described above, contained copurified DNA. To sequence these *E. coli*-derived genomic fragments, the RNP was first incubated in 0.5 X rCutSmart Buffer (NEB) at 37°C for an hour to allow any potential cleavage reactions to proceed to completion. The products were cleaned up using PCR Purification Kit (QIAGEN, 28104) and eluted in water. The cleaved *E. coli* genomic DNA was prepared for next-generation sequencing as previously described (94, 95). Briefly, DNA was treated with terminal deoxynucleotidyl transferase (TdT) (NEB, M0315) for 3′-tailing with dATP for 30 min at 37°C before inactivating the TdT at 70°C for 5 min. An anchored primer (ssExt_pT9_anchor, containing Illumina adapter sequence, nine thymines and a non-thymine) was annealed to the TdT extended products, and the complementary strand was created using Klenow Fragment (3′→5′ exo-) (NEB, M0212). This product was cleaned up using the PCR Purification kit and eluted again in water. Finally, Illumina adapters (oligos ssd_adapt_top and ssd_adapt_bottom) were ligated to the 3′ end of the complementary strand using Blunt/TA Ligase Master Mix (NEB, M0367). All products were indexed by PCR amplification and sequenced on an Illumina MiSeq with Read1 300 cycles, Index1 8 cycles and Index2 8 cycles. Obtained reads were analyzed using a custom script (available on GitHub and archived on Zenodo(96)).

### TasR RNP purification for biochemical and structural analysis

To simplify biochemical and structural analysis, we wanted to purify a TasR RNP that only contained a single guide tigRNA sequence. To this end, a single expression vector containing a T7 promoter, TasR protein coding sequence, and a downstream TIGR array minimized to 3 units with identical spacers to tigRNA1, followed by the natural TIGR array terminator (pMW363) was transformed into *E. coli* BL21(DE3). Cells were grown at 37°C in TB supplemented with 0.5% (w/v) glycerol, 0.2% (w/v) α-lactose monohydrate, 0.05% (w/v) glucose, 2 mM magnesium chloride (TB-based autoinduction media), and 50 μg/L ampicillin. The temperature was reduced to 18°C during mid-log phase, and cells were grown for another 16 hr. Cells were harvested and suspended in TasR lysis buffer (50 mM Tris-HCl pH 7.4, 500 mM NaCl, 5% glycerol, 5 mM beta-mercaptoethanol) supplemented with 0.5 mM PMSF. Cells were lysed using a LM20 microfluidizer device (Microfluidics), and cleared lysate was bound to Strep-Tactin Superflow Plus resin (Qiagen). Resin was washed first with lysis buffer and then with 20 mM HEPES-KOH pH 7.9, 150 mM potassium chloride before elution with the same buffer supplemented with 5 mM desthiobiotin. Fractions containing protein and RNA were concentrated in an Amicon Ultra-4 centrifugal concentrator (30000 MWCO; Millipore) until OD260 nm = 23.1 and OD280 nm = 17.3. Based on theoretical extinction coefficients, yields of nucleic acids extracted using phenol/chloroform, and protein band intensities on SDS-PAGE, we estimated this to correspond to 51 μM of a TasR_2_:tigRNA1_1_ 2:1 complex.

### In vitro cleavage assays

Double-stranded DNA (dsDNA) substrates were produced by PCR amplification of pTarget plasmids or by annealing of labelled synthesized DNA fragments containing the target sites. Cy3 and Cy5-conjugated DNA oligonucleotides (IDT) were used as primers to generate the labeled dsDNA substrates. Single-stranded DNA (ssDNA) and single-stranded RNA (ssRNA) substrates were ordered as Cy3 and Cy5-conjugated oligonucleotides (IDT). Through annealing of the ssRNA and its complement ssRNA, double-stranded RNA (dsRNA) substrates were prepared. For generation of 100-bp Fluorescein/Cy5 labelled substrates used in Figs. 3 and 4E and fig. S7C, D, ‘C’ and ‘W’ oligos (table S3) were annealed and diluted to 1 nM in PCR reactions with primers label_targets_F and label_targets_R (table S3), and reactions products were purified using SPRI beads. For the cleavage assays shown in Fig. 4B-D and fig. S7A, B, E, reaction mixtures (10 μl) contained 100 ng of substrate, 10 μM of TIGR RNP, 10 mM Tris pH 8.0, 50 mM NaCl, and 5 mM MgCl_2_. For Fig. 4B, instead of TIGR RNPs, the apoproteins were incubated with 20 μM synthesized 36-nt tigRNA (IDT) or *in vitro* transcribed tigRNA. For reactions using *in vitro* transcribed tigRNA, 5 units of Shrimp Alkaline Phosphatase (NEB, M0371) was added into the reaction mixture to dephosphorylate *in vitro* transcribed tigRNA. Assays were allowed to proceed at 37°C for 2 hours. Reactions were then treated with RNase A (Qiagen, 19101) and Proteinase K (NEB, P8107) and purified using a Monarch PCR & DNA Cleanup Kit (NEB, T1030). RNA substrate cleavage assays were carried out without RNase A treatment. For screening metal ions, the indicated metal chloride was added instead of MgCl_2_.

For the cleavage assays shown in Figs. 3C, 4E and figs. S7C-D, and for the cryo-EM structure, the TasR RNP only contained tigRNA1. Reaction mixtures (10 μl) contained 20 nM of labelled DNA substrate, 200 nM of TIGR RNP, 20 mM HEPES-KOH pH 7.9, 150 mM KCl, and 5 mM MgCl_2_. Reactions were incubated at 42°C for 1 hour before treatment with 0.8 U proteinase K (NEB) at 37°C for 15 min. Reaction products were not further purified.

All reaction mixtures were resolved by denaturing gel electrophoresis on 10% TBE-Urea polyacrylamide gels (Thermo Fisher Scientific, 12010126) at 400 V and imaged using a ChemiDoc (Bio-RAD).

### In vitro TAM screen

After *in vitro* cleavage assay with 25 ng of pTarget containing an 8N motif upstream of the PSP1 sequence, the cleaved DNA was extracted using the PCR Purification kit and adaptors were ligated using an NEBNext Ultra II DNA Library Prep Kit for Illumina (NEB, E7645) using the NEBNext Adaptor for Illumina (NEB, E7337A). Following adaptor ligation, cleaved products were amplified specifically using one primer specific to the TAM library backbone and one primer specific to the NEBNext adaptor with a 12-cycle PCR using NEBNext High Fidelity 2X PCR Master Mix (NEB, M0541) with an annealing temperature of 63°C, followed by a second 18-cycle round of PCR to further add the Illumina i5 adaptor. Amplified libraries were gel extracted and subjected to single-end sequencing on an Illumina MiSeq with Read1 80 cycles, Index1 8 cycles and Index2 8 cycles. TAMs were extracted, and an enrichment score for each TAM was calculated by filtering for all TAMs present more than once and normalizing to the TAM frequency in the input library. A position weight matrix based on the enrichment score was generated and the results were visualized based on this position weight matrix using a custom script (available on GitHub and archived on Zenodo(97)).

### Plasmid loss assay

*E. coli* Stbl3 was co-transformed with either pMW364 + pMW362, pMW364 + pMW27, pMW366 + pMW362, or pMW370 + pMW362 and plated on LB agar + ampicillin + chloramphenicol. Three colonies of each were grown overnight in LB + ampicillin + chloramphenicol before dilution into either LB + ampicillin + 0.2% (w/v) glucose or LB + ampicillin + 0.2% (w/v) arabinose and growth at 37°C for 18 hr. 10-fold serial dilutions in PBS were then spotted onto LB agar + ampicillin and LB agar + ampicillin + chloramphenicol. Plasmid maintenance was calculated as colony forming units on ampicillin + chloramphenicol divided by colony forming units on ampicillin.

### Conjugation efficiency assay

Three independent colonies of *E. coli* Stbl3 harboring pMW364 were grown overnight in LB + ampicillin + 0.2% arabinose. Three independent colonies of *E. coli* β2163 (F− RP4-2-Tc::Mu Δ*dapA::(erm-pir)*, strain a gift from Rita Monson, University of Cambridge) (98) harboring pMW373 or pMW374 were grown overnight in LB + chloramphenicol + 0.3 mM diaminopimelic acid. Donor and recipient strains were pelleted, washed twice with PBS, then mixed with equal ratios before incubating on 0.45 μm filters placed on LB agar + 0.3 mM diaminopimelic acid + 0.2% arabinose. After incubation at 37°C for 3.5 hr, bacteria were recovered from filters by washing with LB, and 10-fold serial dilutions in PBS were spotted onto LB agar + ampicillin and LB agar + ampicillin + chloramphenicol. Conjugation efficiency was calculated as colony forming units on ampicillin + chloramphenicol divided by colony forming units on ampicillin.

### Phage defense assay

*E. coli* Stbl3 harboring pMW364 or pMW382 was grown overnight in LB + ampicillin before dilution into LB + ampicillin + 0.2% arabinose and growth to saturation. Cultures were mixed with LB + 0.7% (w/v) top agar + ampicillin + 0.2% arabinose and poured over LB agar + ampicillin. 3 μL of 10-fold serial dilutions of phages ZL19 and T5 were spotted onto the top agar and incubated at 37°C overnight.

### Cryo-EM sample preparation and data collection

To assemble a TasR TIGR RNP product complex, TasR tigRNA1 RNP (16 μM) was mixed with a 60-bp DNA target created by annealing two complementary unlabelled 60-nt DNA oligonucleotides (8 μM) in a buffer containing final concentrations 29 mM HEPES-KOH pH 7.9, 149 mM KCl, 5 mM MgCl_2_. The mixture was incubated at 42°C for 1 hour then placed on ice for 3 hours. The reaction mixture was then applied to a glow-discharged (60 s at 25 mA) Cu300 R1.2/1.3 holey carbon grid (Quantifoil) mounted in the chamber of a Vitrobot Mark IV (Thermo Fisher Scientific) maintained at 12°C and 100% humidity. Grids were blotted using Ø55 grade 595 filter paper (Ted Pella) for 4 s and plunged into liquid ethane. Cryo-EM data were collected using the Thermo Scientific Titan Krios G3i cryo TEM at MIT.nano using a K3 direct detector (Gatan) operated in super-resolution mode with 2-fold binning, and an energy filter with slit width of 20 eV. Micrographs were collected automatically using EPU in AFIS mode at 130,000× magnification with a real pixel size of 0.663 Å. 14,257 movies were collected, using an exposure time of 1.09 s, fractionated into 40 frames and a flux of 17.82 e-/pix/s giving a total fluence per micrograph of 45.4 e-/Å2.

### Cryo-EM data processing

Movies were corrected for motion using the RELION implementation of MotionCor2, with 6x4 patches and dose-weighting. CTF parameters were estimated using CTFFIND-4.1 (99). Particles were picked using cryoSPARC 4.6 on denoised micrographs using the blob picker and a particle diameter between 60 and 120 Å (100). Picked particles were classified in 2D, and 4 classes representing common views were then used as templates for a second round of picking on the denoised micrographs. Particle coordinates from this second round of picking were then imported into RELION-5.0 (101) and used to extract 10,065,225 particles with a box size of 256 pixels binned during extraction to 96 pixels, and 1.744 Å/pix. An initial model was generated from 540,459 particles selected from 1500 micrographs by 2D classification of particles picked within RELION using Topaz (102). The apparently C2-symmetric model was aligned to the C2 symmetry axis and C2 symmetry was applied. One round of 3D classification with C1 symmetry, using this initial model with a 20 Å low-pass filter, Blush regularization, a 120 Å spherical mask, a 10 pixel translational search range, --iter 25 --tau2_fudge 4 --K 4 --fast_subsets --maxsig 500, was used to select 3,670,750 promising particles. A second round of 3D classification with 1.8 degree local angular searches with C2 symmetry relaxation, Blush regularization, a 120 A diameter spherical mask, --iter 25 --tau2_fudge 4 --K 4, yielded 763,089 particles with well-defined features. These particles were reextracted from the micrographs motion-corrected using RELION with a 256 pixel box binned to 172 pixels, using the defocus values determined by the patch CTF job in cryoSPARC. 3D refinement in RELION used a 20A low-pass filtered reference, a soft mask around the whole complex, solvent-flattened FSCs, Blush regularization, 5 pixel translational searches, and angular searches initiated with 7.5 degree global sampling, with local searches (with C2 symmetry expansion) from 1.8 degrees sampling onwards. These parameters yielded a 3.28 Å resolution map with clear density differences between the pseudosymmetry-related spacer A and spacer B heteroduplexes, suggesting the symmetry was broken effectively by the symmetry expansion procedure. Particles were further improved using Bayesian polishing, using the trained parameters --s_vel 1.0095 --s_div 7260 --s_acc 2.925 and extracting using a 288 pixel box and binned to 192 pixels, 0.981 Å/pix. A final refinement (with the same parameters as above except with 3 pixel translational searches) produced an isotropic, symmetry-broken map at 3.05 Å resolution with features consistent with the estimated resolution. All resolutions are reported using the gold-standard Fourier Shell Correlation with 0.143 cutoff.

### Cryo-EM model building

An AlphaFold2 model of the TasR ORF was docked into one corresponding protomer in the cryo-EM density and fitted using ISOLDE and adjusted in Coot (103, 104). An idealised RNA/DNA duplex was placed into the helical density proximal to the fitted protomer, and was identified as the spacer A duplex by the match of the larger bases to purine positions within spacer A or its target, and by clear differences in the pseudo-C2-related equivalent density. This fitting set the register for interpreting the rest of the nucleic acids in the complex. The upstream and downstream double-stranded DNA was fit as idealized B-form helices before adjusting to the density and mutation to the in-register sequence. The box C and box D motifs of the tigRNA were then added to spacer A, before fitting the same model (and protein model) to the pseudosymmetry-related density and altering the sequence to spacer B. Continuous density linking spacer to B was interpreted as the loop repeat, identifying the pseudosymmetrically related density as the edge repeat, however density towards the 5′ and 3′ ends of the edge repeat was difficult to interpret in detail. The model was refined first using ISOLDE, then with Phenix real_space_refine (105). Cryo-EM data statistics and model statistics can be found in table S2.

### Mammalian cell culture and transfection

Mammalian cell culture experiments were performed in the HEK293FT cell line (Thermo Fisher, R70007) grown in Dulbecco’s modified Eagle medium with high glucose, sodium pyruvate, and GlutaMAX (Thermo Fisher, 10569010), and additionally supplemented with 10% fetal bovine serum (VWR Seradigm). Transfections were performed with Lipofectamine 3000 (Thermo Fisher, L3000015) in 96-well plates unless otherwise noted. Cells were plated at approximately 2.0 X 10^4^ cells per well 16-20 hours before transfection to ensure 90% confluency at the time of transfection. For each well on the plate, transfection plasmids (100 ng in total) were combined with OptiMEM Reduced Serum Medium (Thermo Fisher, 31985062) to a total volume of 5 μl and mixed with 0.2 μl of P3000 reagent. Separately, 5 μl of OptiMEM was combined with 0.3 μl of Lipofectamine 3000 reagent. Plasmid and Lipofectamine solutions were then combined, incubated at room temperature for 10 minutes and pipetted onto cells.

### Human genome cleavage assay

For human genome cleavage assays, 2.0 X 10^4^ of HEK293FT cells in 96-well plates were co-transfected with combinations of TaTasR or ParTasR expression plasmid (50 ng) and tigRNA expression plasmid (50 ng). After 3 days of incubation at 37°C, the supernatant was removed and cells were resuspended in 40 μL QuickExtract DNA Extraction Solution (Lucigen, QE09050) and cycled at 65°C for 15 minutes, 68°C for 15 minutes, then 95°C for 10 minutes to lyse cells. 2 μL of lysate was used as the template for each 12.5 μl-PCR reaction. Target sites were amplified with NEBNext High-Fidelity 2X PCR Master Mix (NEB) under the following thermal cycling conditions: 1 cycle, 98°C, 30 seconds; 15 cycles, 98°C, 10 seconds, 65°C, 20 seconds, 72°C, 30 seconds; 1 cycle, 72°C, 30 seconds; 4°C hold. 1 μL of this first PCR product was used for the template for each 10-μl second PCR reaction: 1 cycle, 98°C, 30 seconds; 15 cycles, 98°C, 10 seconds, 63°C, 20 seconds, 72°C, 30 seconds; 1 cycle, 72°C, 30 seconds; 4°C hold (total 30 cycles for first and second PCR reactions). Amplicons were sequenced on an Illumina MiSeq with Read1 300 cycles, Index1 8 cycles and Index2 8 cycles. Indel efficiency was quantified using the established CRISPResso2 pipeline.

### TIGR association with *parB*-like gene

Genes located within a 5-kb vicinity of the *tas* genes were extracted, translated, and clustered at 50% sequence identity and 70% coverage using MMseqs2 (75). We manually inspected the most abundant genes near *tas* genes, focusing on those operonized with *tas* genes. Among these, a *parB*-like gene, termed *tasParB*, was consistently observed in operons with *tasA* genes. Alignments of several loci indicated that the operon structure is conserved, while flanking regions display substantial diversity in gene composition (fig. S14). Manual inspection revealed the presence of dual-repeat TIGR arrays upstream, downstream, and sometimes between the *tasParB* and *tasA* genes. These arrays contain nucleotide spacers flanked by 10-nucleotide repeats, including edge and loop repeats. Structural multimer assembly was performed using AlphaFold3 webserver (106) to model complexes comprising two TasA and two TasParB proteins (fig. S14D). TasA proteins are predicted to form a head-to-head dimer, a feature distinct from other Tas protein family members. TasParB proteins are predicted to dimerize via cofolding of their C-terminal regions, a characteristic also observed in other ParB-like domain systems (107). Functional annotations of TasParB were derived using HHpred (108), which identified matches to various ParB domains (54, 109), including ParB-like CTPases (110).

## Supplementary Material

Table S3

Supplemental Material

Data S1

Data S2

Table S1
