# Supplementary material for: TIGR-Tas: A Family of Modular RNA-Guided DNA-Targeting Systems in Prokaryotes and Their Viruses
Source: Science. Author manuscript; Available in PMC 2025 May 2. (PMC12045711; doi:10.1126/science.adv9789)
Supplement: Supplemental Material [file NIHMS2071934-supplement-Supplemental_Material.pdf]

Science
AAAS

Supplementary Materials for
TIGR-Tas: A Family of Modular RNA-Guided DNA-Targeting Systems in Prokaryotes and Their Viruses

Guilhem Faure[1-5]†, Makoto Saito[1-5]†, Max E. Wilkinson[1-5]†, Natalia Quinones-Olvera[1-5], Peiyu Xu[1-5], Daniel Flam-Shepherd[1-5], Stephanie Kim[1-5], Nishith Reddy[1-5], Shiyou Zhu[1-5], Lilia Evgeniou[1-6], Eugene V. Koonin[7], Rhiannon K. Macrae[1-5], and Feng Zhang[1-5]*

Corresponding author: zhang@broadinstitute.org

The PDF file includes:

Figs. S1 to S14
Tables S2
References 112 - 113

Other Supplementary Materials for this manuscript include the following:

Table S1, S3
Data S1 to S2

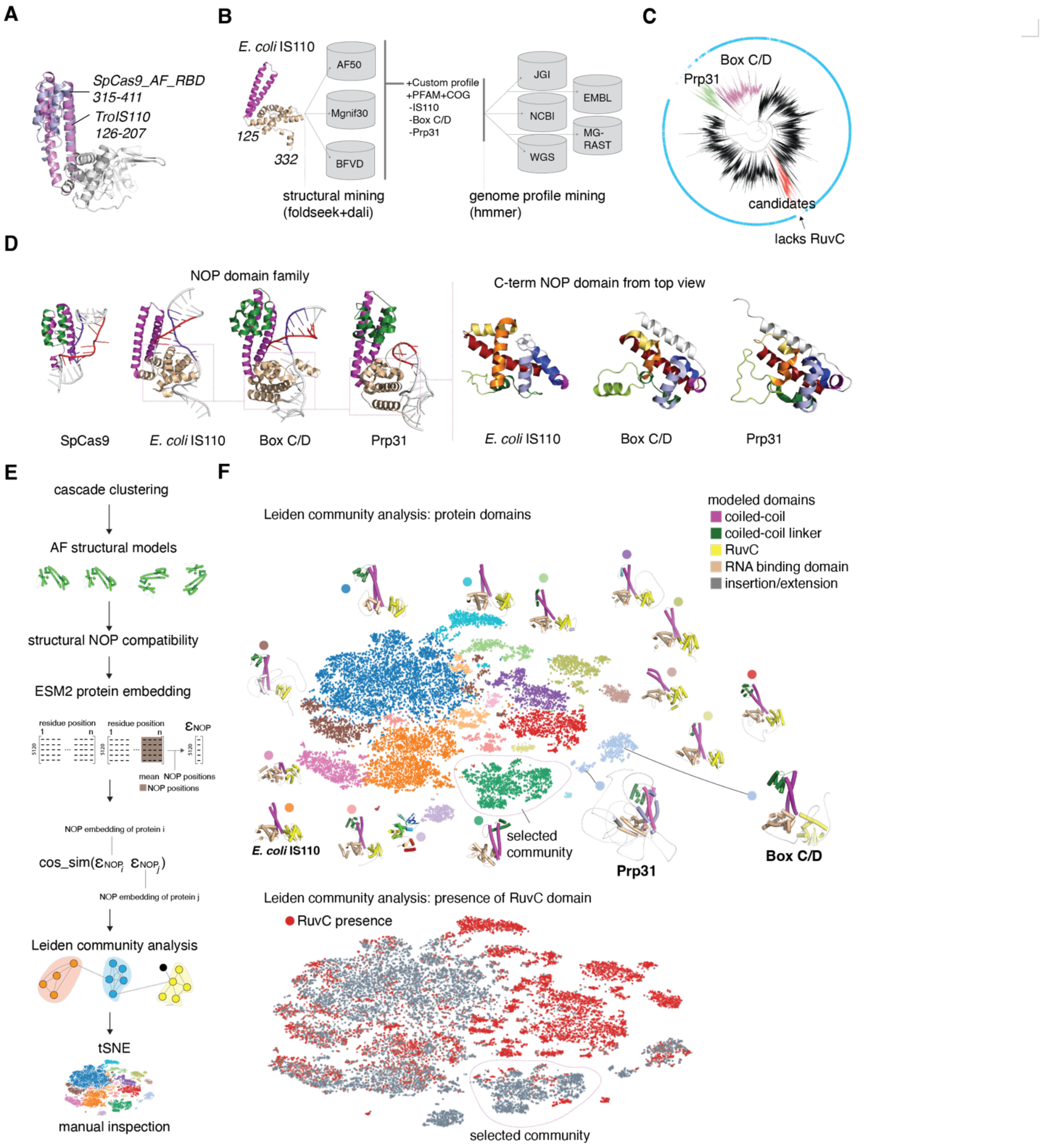

Fig. S1. Pipeline for TIGR system discovery

(A) Structural superimposition of the RNA-binding domain (RBD) from an AlphaFold model of SpCas9 (dark purple) with a region of *Tropicimonas sp.* IS110 (TroIS110, pink). The best-aligned regions are indicated with coordinates for SpCas9 and TroIS110. **(B)** Structural mining pipeline starting from the RBD of *E. coli* IS110 (EcIS110), including the region structurally similar (purple) to the SpCas9 RBD extended to the C-terminal domain of IS110 (wheat). Structural mining was performed using Foldseek and DALI across databases: AlphaFold clustered at 50% sequence identity (AF50), the EMBL-EBI metagenomic database folded by

ESMFold (clustered at 30% sequence identity, Mgnify30), and a protein-virus database folded by AlphaFold (BFVD). This process revealed structural similarities to the Nop domain family (see panels **C and D**). 12 domain profiles related to the Nop domain-containing family were used for genome profile mining using hmmsearch across JGI, NCBI, WGS, EMBL, and MG-RAST database. **(C)** Phylogenetic tree of Nop domain candidates obtained through structural mining. The outer ring indicates the presence of the RuvC domain detected by structural comparison (regardless of catalytic activity) to EcIS110 RuvC using Dali. Box C/D Nop domains are highlighted in purple. Two clades lack RuvC domains, corresponding to Prp31 (green) and an unknown clade (red). Proteins from the unknown clade were converted into a profile for further genomic profile mining. **(D)** Structural comparison of the RBD of SpCas9 (interacting with its guide RNA), EcIS110 (seed region used for mining and interacting with bridge RNA), and members of the Nop domain family, including box C/D snoRNAs and Prp31. Shared structural features are highlighted with matching colors. The right panel provides an in-depth comparison of the C-terminal region of the Nop domain, shared by EcIS110, box C/D, and Prp31. Distinct helices are colored to emphasize structural similarities. **(E)** Genomic profile mining pipeline. Hits from genomic mining were clustered at varying sequence identity thresholds down to 50%. The representative sequences were folded using AlphaFold2. Candidates were filtered to retain only those with structural similarity to the *E. coli* Nop domain seed using Dali. Protein embeddings were generated using the ESM2 15B model, and embeddings were extracted only for Nop-specific positions. Nop embeddings for each candidate were average and compared via cosine similarity. Leiden community detection was applied to identify families of Nop domains. Leiden communities were mapped onto a t-SNE projection of the Nop embeddings. **(F)** Leiden communities were mapped onto a t-SNE projection of the Nop embeddings. Each community was manually inspected, examining structural models and full protein features. RuvC nucleases structural domains (regardless of catalytic activity) are shown in yellow, Nop domain regions in magenta (N-terminal coiled coil helices), green (coiled coil linker), and wheat (C-terminal). Extensions are depicted in grey. The box C/D and Prp31 community are shown. A large group near Prp31 and box C/D, largely lacking RuvC domains, was selected for further investigation.

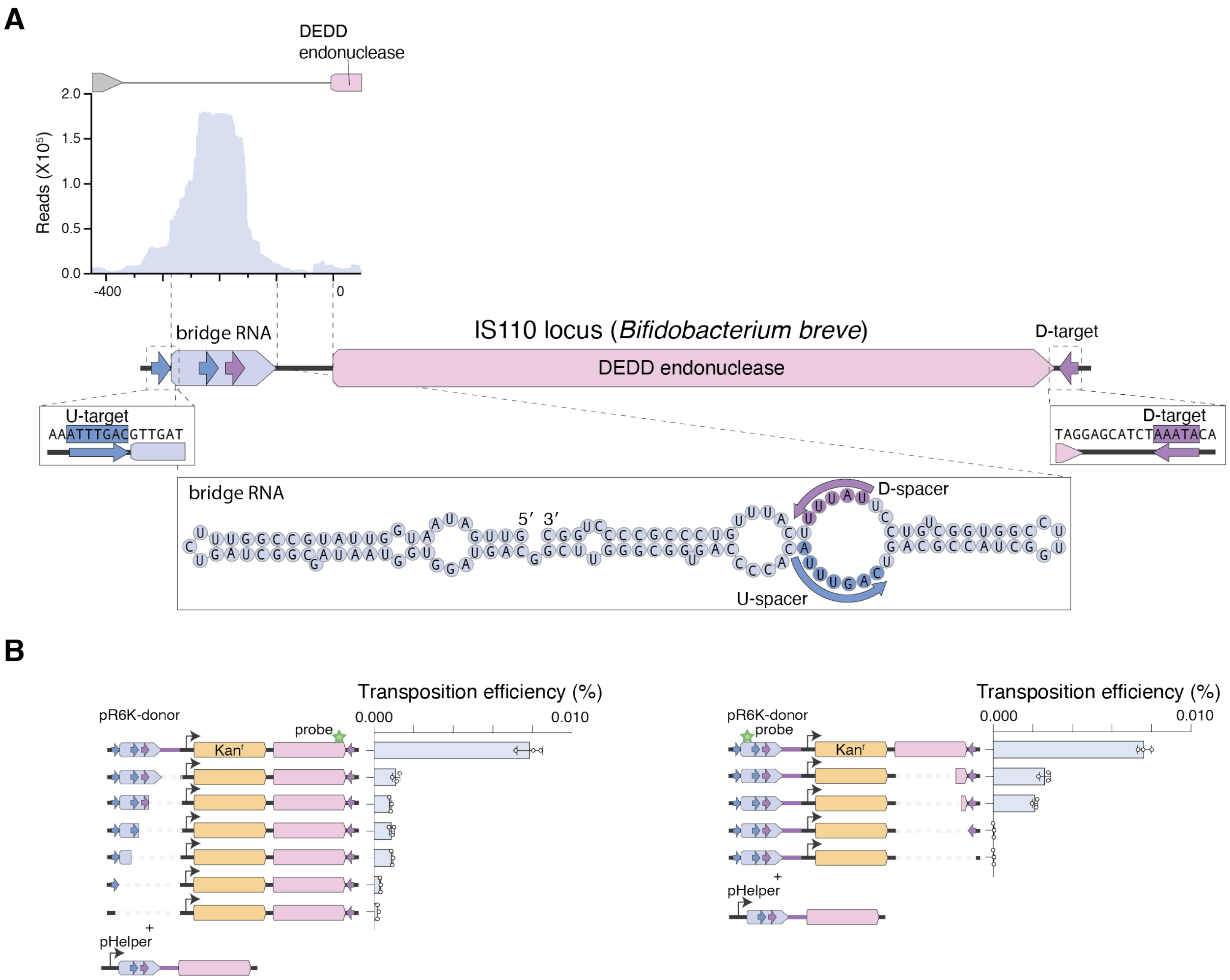

Fig. S2. IS110 is an RNA-dependent insertion element

(A) RNA-seq analysis of the *Bifidobacterium breve* IS110 locus reveals reads mapping to the upstream region of the IS110 gene. The schematic of the locus highlights the IS110 gene (pink) and the associated ncRNA upstream. The ncRNA contains two distinct spacers, termed D-spacer and U-spacer, embedded within the loop of a predicted hairpin structure. These spacers match complementary sequences located upstream (U-target) and downstream (D-target) of the IS110 gene, suggesting RNA-mediated targeting. **(B)** Disruption of either the ncRNA or the protein integrity significantly reduces transposition efficiency. The graph illustrates the quantitative impact on transposition rates under various conditions, underscoring the requirement of both the ncRNA and the IS110 protein for optimal transposition activity. Probe used for the efficiency quantification by ddPCR is annotated as star.

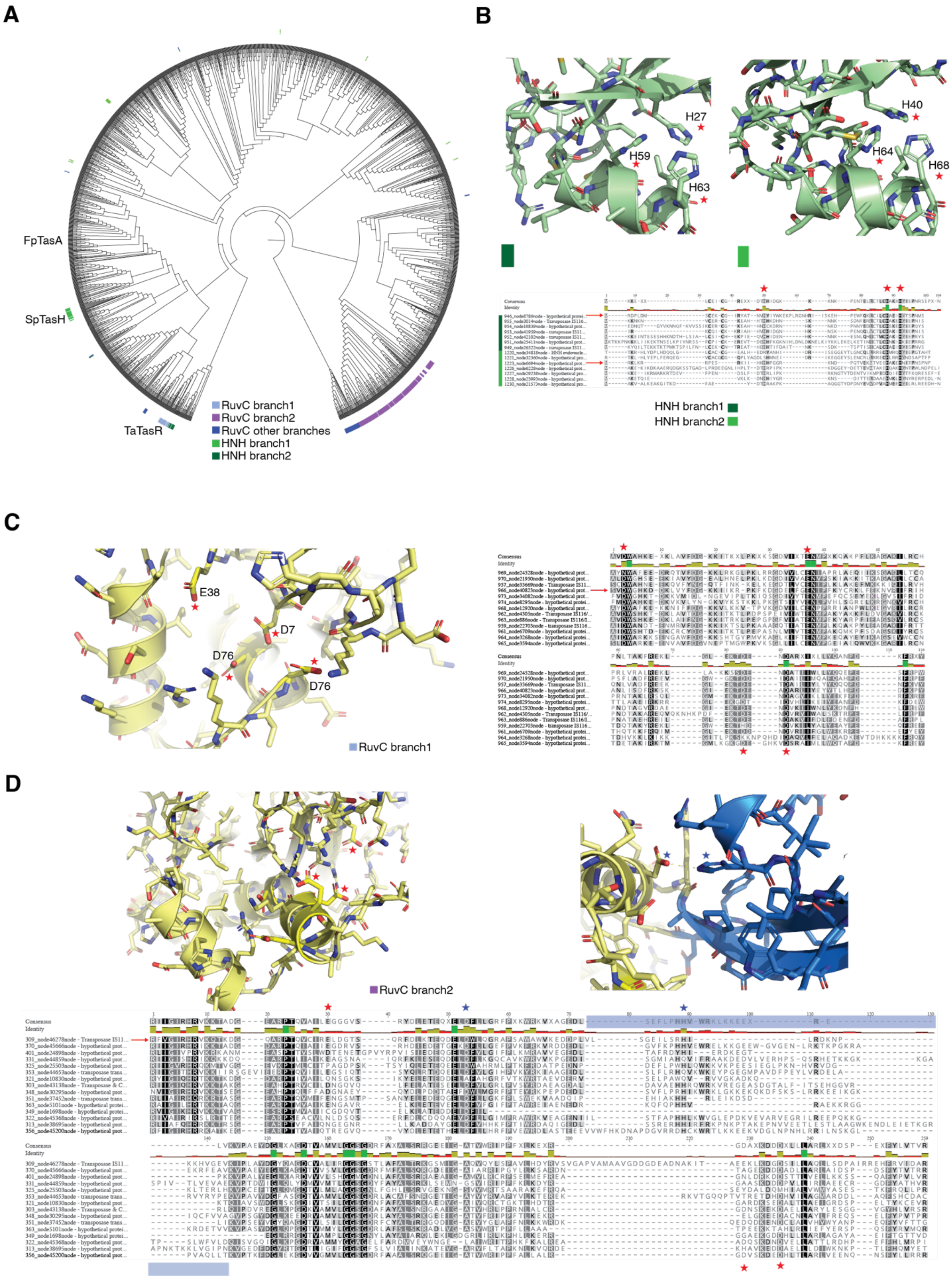
A
B
FpTasA
SpTasH
TaTasR
RuvC branch1
RuvC branch2
RuvC other branches
HNH branch1
HNH branch2
H27
H59
H63
H40
H64
H68
HNH branch1
HNH branch2
C
E38
D7
D76
D76
RuvC branch1
D
RuvC branch2

Fig. S3. Tas nuclease diversity

(A) Phylogenetic tree of Tas Nop domains. The outer ring indicates the presence of nuclease domains: light blue for group 1 (active RuvC), purple for group 2 (inactive RuvC), dark blue for additional RuvC domains (sparse RuvC across the tree, and a small clade with inactive RuvC), and light/dark green for two distinct groups of HNH nucleases. **(B)** HNH nuclease catalytic site. Structural models highlight the conserved catalytic residues for both HNH groups. Below, a sequence alignment shows conserved positions across HNH nuclease sequences, with red stars marking the catalytic residues. The red arrow indicates which sequences were used for the structural models shown above. **(C)** Active RuvC catalytic sites. Structural models display the positions of conserved catalytic residues within active RuvC domains. A sequence alignment below highlights the catalytic residues, marked with red stars. **(D)** Inactive RuvC domains. Structural models show the positions of non-conserved residues within catalytically inactive RuvC domains. A zoomed-in view reveals a conserved structural insertion (in blue) within the RuvC domain, including a residue forming a salt bridge with RuvC and the insertion. Sequence alignment indicates the conserved residue positions with stars, and the insertion sequence is shown in blue.

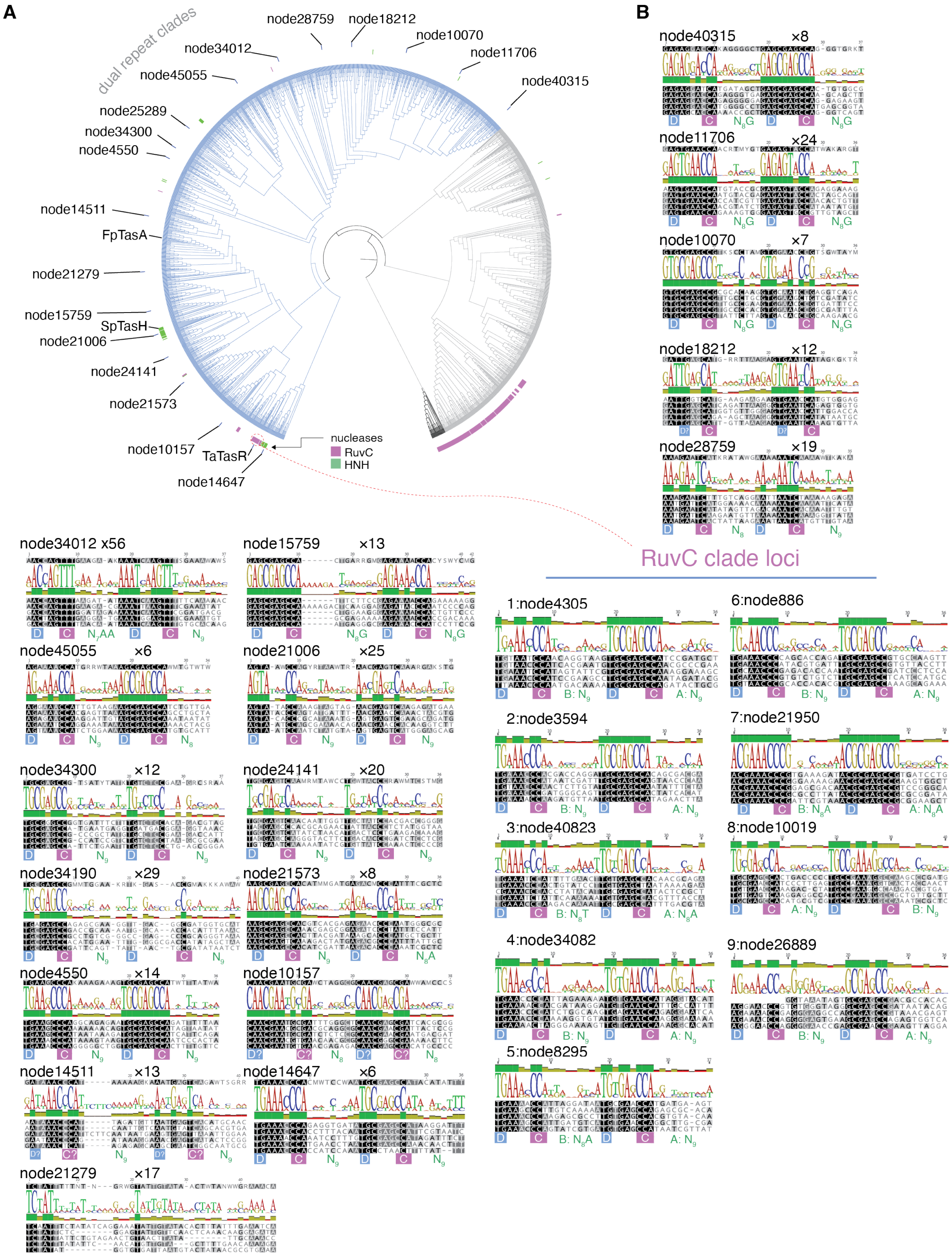
A
B
dual repeat clades
node28759
node18212
node10070
node11706
node40315
node34012
node45055
node25289
node34300
node4550
node14511
FpTasA
node21279
node15759
SpTasH
node21006
node24141
node21573
node10157
TaTasR
node14647
nucleases
RuvC
HNH
node40315 ×8
node11706 ×24
node10070 ×7
node18212 ×12
node28759 ×19
RuvC clade loci
node34012 x56
node15759 ×13
node45055 ×6
node21006 ×25
node34300 ×12
node24141 ×20
node34190 ×29
node21573 ×8
node4550 ×14
node10157
node14511 ×13
node14647 ×6
node21279 ×17
1:node4305
2:node3594
3:node40823
4:node34082
5:node8295
6:node886
7:node21950
8:node10019
9:node26889

Fig S4. Diversity of dual repeat TIGR arrays
(A) Phylogenetic tree of the Nop domain in the Tas family. The inner ring indicates the presence and type of nuclease: green for HNH and blue for RuvC. Nodes labelled around the tree correspond to loci manually inspected for dual repeat arrays; alignments are shown in panel **B**. Blue branches denote association with dual repeat arrays. Loci coordinates and sequences for each node are listed in Supplementary Table 1. FpTasA, TaTasR, SpTasH are indicated in the tree. **(B)** Alignment of dual repeat arrays. Representative alignments of repeat arrays from different loci (labelled in the tree panel **A**) are shown, each consisting of edge repeat, spacer A, loop repeat, and spacer B. Typically, five repeats are displayed per array. WebLogos above each alignment highlight the conserved positions of repeats and spacers, showcasing sequence conservation and variability across arrays. Putative box C and box D motifs are annotated along with spacer lengths. For RuvC-clade arrays, spacers are annotated as 'A' if the preceding repeat is predicted to be an edge repeat by similarity to the TaTasR TIGR array. The repeat arrays of TaTIGR are shown in Fig. 1C. The first and last repeat arrays of FpTIGR and SpTIGR are shown in fig. S5A.

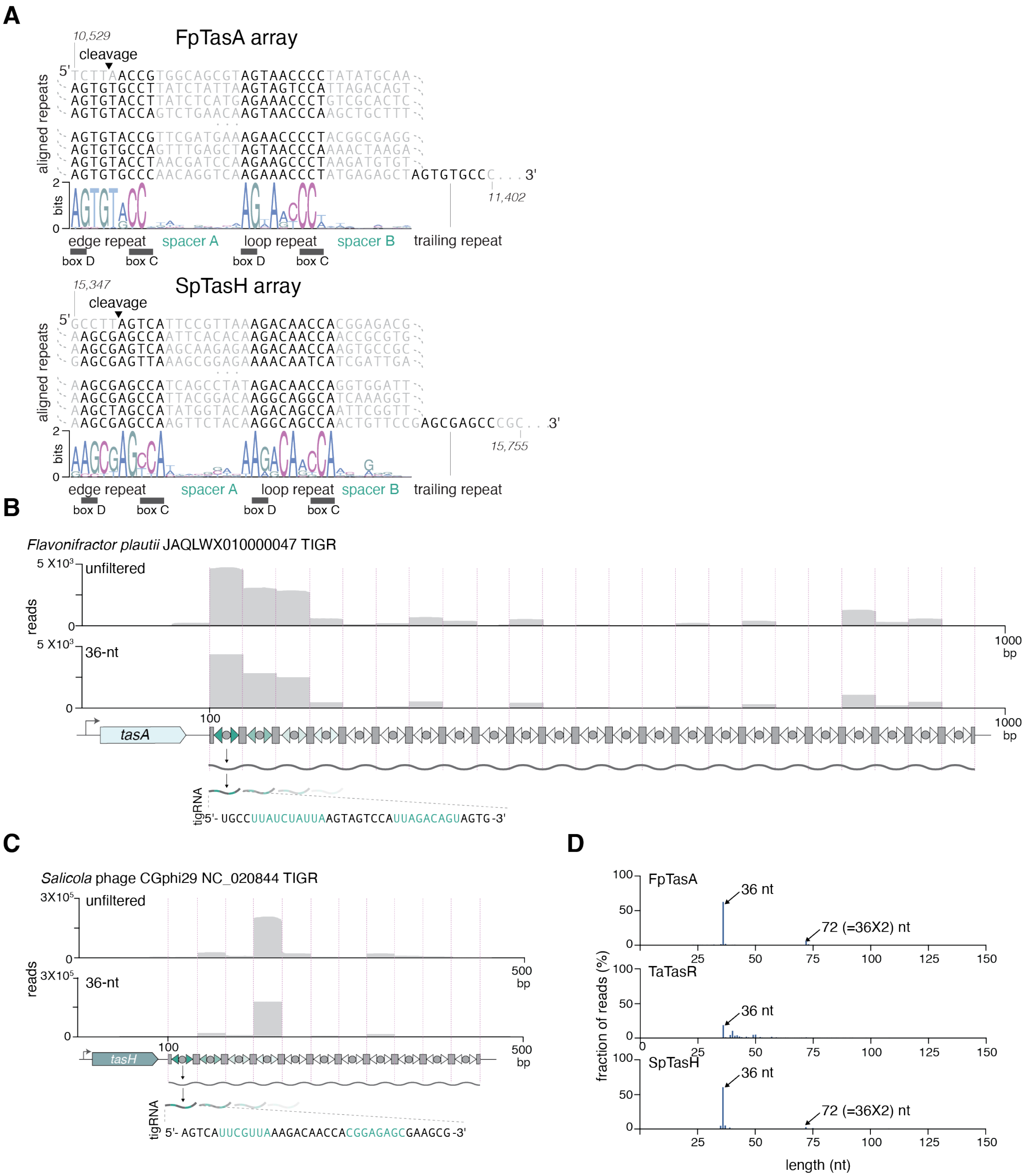

Fig S5. Small RNA-Seq analysis of TIGR arrays

(A) Alignment of repeats from the FpTIGR and SpTIGR arrays shown in panels B and C. Conserved regions corresponding to edge and loop repeats are shown in bold, and while variable regions represent spacers A and B. Note that here we define spacers by their positions relative to the conserved box C and box D motifs. Therefore, the FpTIGR repeats end with a non-conserved nucleotide (CCN box C) that is strictly still part of the repeat, whereas the SpTIGR spacers end with a conserved A (N8A spacer) that is outside of the AG box D and we define as being part of the spacer. Below the alignment, a WebLogo illustrates sequence conservation across the array.

(B,C) Small RNA-seq of RNA pulled down with (B) *Flavonifractor plautii* TasA (FpTasA) or **(C)** *Salicola phage* TasH (SpTasH) mapped to the FpTIGR and SpTIGR loci, respectively. Top: All reads mapped to **(B)** FpTIGR or **(C)** SpTIGR array expressed in *E. coli*. Bottom: Mapping of 36 nt reads reveals that the pre-tigRNA transcript is processed into distinct 36 nt tigRNA units. **(D)** Distribution of small RNA-seq read lengths from RNA pulled-down with Tas protein. Histograms show the distribution of read lengths (after adapter trimming) from small RNA-seq data of TIGR systems expressed in *E. coli*. Data are shown for FpTIGR, *Thermoproteota archaeon* TIGR (TaTIGR), and SpTIGR. The fraction of reads (%) is normalized to the total number of reads, highlighting a prominent peak at 36 nt, indicative of processed tigRNA units. A peak at 72 nt, a multiple of 36, is indicative of a partially processed tigRNA.

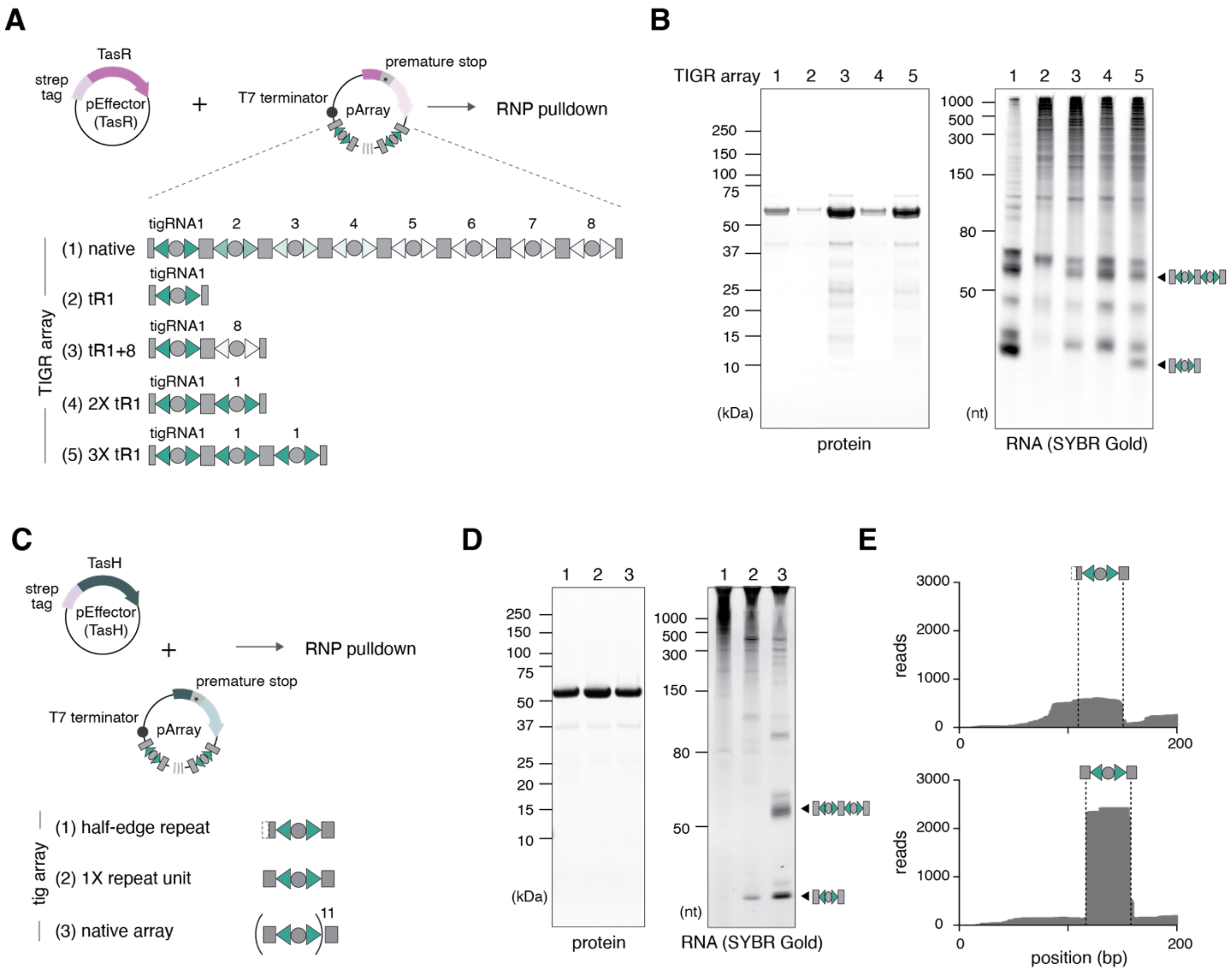

Fig S6. Repeat unit number requirement and minimization of TIGR array
(A) Schematic of *TaTIGR* expression constructs. Diagram depicting five TIGR array plasmids used for expressing *TaTasR* and associated tigRNAs. Variations include different numbers and identities of repeat units. In pEffector, TasR has a Twin-Strep-SUMO tag. **(B)** Validation of TaTIGR RNP assembly. Representative SDS-PAGE gel (left) shows the expression of TaTasR protein, and a denaturing PAGE gel (right) verifies the presence of tigRNA in RNP complexes purified from *E. coli*. **(C)** Schematic of *SpTIGR* expression constructs. Diagram depicting three TIGR array plasmids used for expressing *SpTasH* and associated tigRNAs, including minimized constructs. **(D)** Validation of SpTIGR RNP assembly. Representative SDS-PAGE gel (left) shows the expression of SpTasH protein, and a denaturing PAGE gel (right) verifies the presence of tigRNA in RNP complexes purified from *E. coli*. **(E)** Small RNA-seq mapping of SpTIGR array constructs. Top: Mapping of small RNA-seq reads to the half-edge repeat construct, showing that 5′ of tigRNA was not processed at the half-edge repeat. Bottom: Mapping of small RNA-seq reads to the single repeat unit construct, demonstrating tigRNA production from a minimal TIGR array configuration.

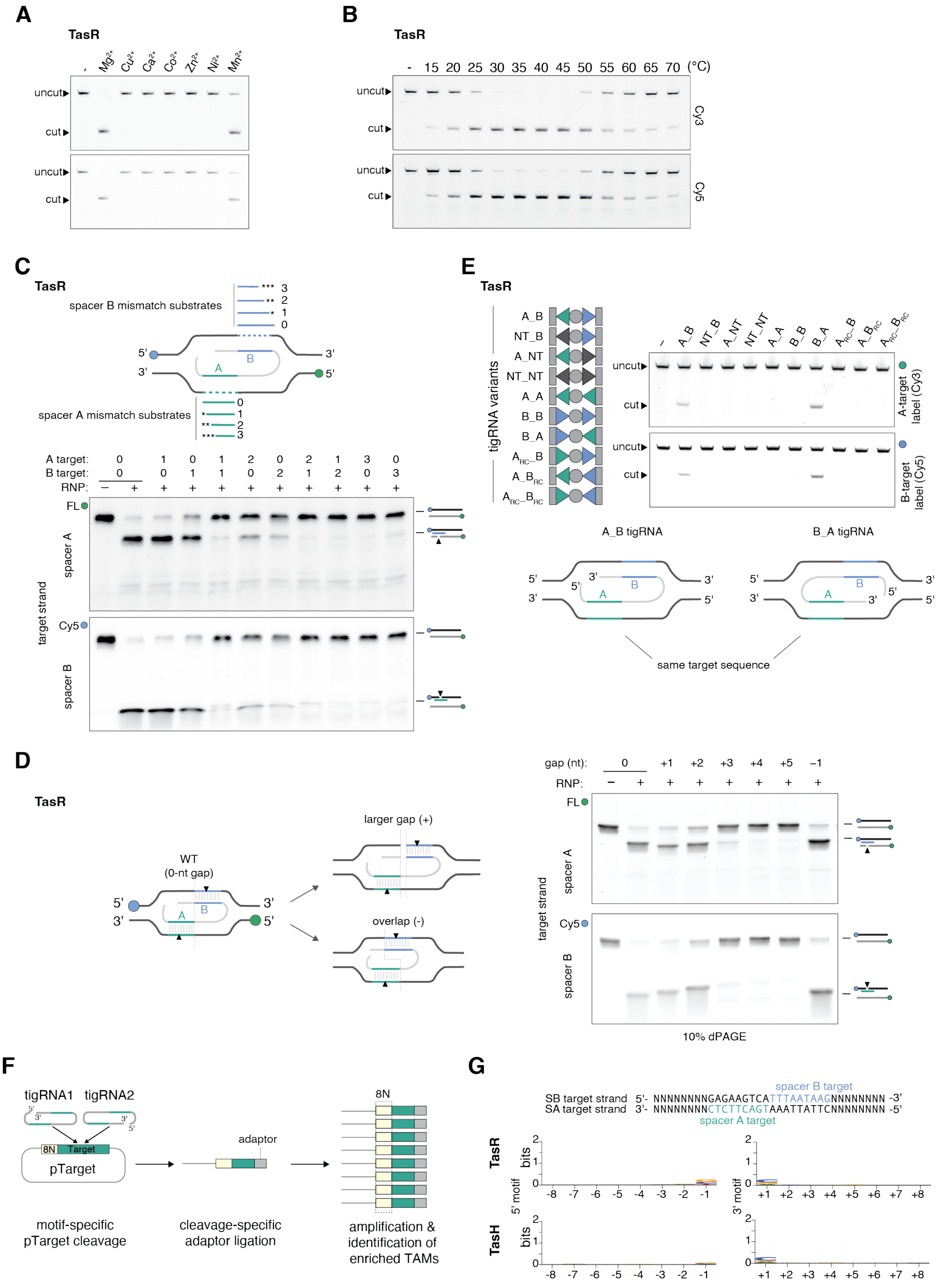

A
TasR
-
Mg2+
Cu2+
Ca2+
Co2+
Zn2+
Ni2+
Mn2+
uncut
cut
uncut
cut
B
TasR
- 15 20 25 30 35 40 45 50 55 60 65 70 (°C)
uncut
cut
Cy3
uncut
cut
Cy5
C
TasR
spacer B mismatch substrates
*** 3
** 2
* 1
0
5'
3'
B
A
3'
5'
spacer A mismatch substrates
0
* 1
** 2
*** 3
A target: 0 0 1 0 1 2 0 2 1 3 0
B target: 0 0 0 1 1 0 2 1 2 0 3
RNP: – + + + + + + + + + +
FL
spacer A
target strand
Cy5
spacer B
E
TasR
tigRNA variants
A_B
NT_B
A_NT
NT_NT
A_A
B_B
B_A
A_RC_B
A_B_RC
A_RC_B_RC
uncut
cut
uncut
cut
A-target label (Cy3)
B-target label (Cy5)
A_B tigRNA
B_A tigRNA
same target sequence
D
TasR
WT
(0-nt gap)
larger gap (+)
overlap (-)
gap (nt): 0 +1 +2 +3 +4 +5 –1
RNP: – + + + + + + +
FL
spacer A
target strand
Cy5
spacer B
10% dPAGE
F
tigRNA1
tigRNA2
8N
Target
pTarget
adaptor
8N
motif-specific pTarget cleavage
cleavage-specific adaptor ligation
amplification & identification of enriched TAMs
G
spacer B target
SB target strand 5'- NNNNNNNNGAGAAGTCATTTAATAAGNNNNNNNN -3'
SA target strand 3'- NNNNNNNNCTCTTCAGTAAATTATTCNNNNNNNN -5'
spacer A target
TasR
TasH
bits
5' motif
3' motif
-8 -7 -6 -5 -4 -3 -2 -1
+1 +2 +3 +4 +5 +6 +7 +8

Fig S7. Biochemical properties of TaTasR
(A) Target dsDNA cleavage by TaTasR in the presence of various divalent metal ions. All subsequent experiments are performed using Mg^{2+}. **(B)** Target dsDNA cleavage by TaTasR at various temperatures. **(C)** Effects of increasing numbers of mismatches to spacer A and/or spacer B on dsDNA cleavage by TaTasR. Each mismatch is a transversion of type A→T, T→A, G→C, or C→G. Both strands of the DNA target are mutated in this experiment. **(D)** Effect of gaps or overlaps in the DNA spacer A/B-matching sequences. These experiments used TaTasR loaded with tigRNA1, which only permits testing of a 1-nt overlap, as further overlaps also introduce mismatches to the guides which independently compromise activity (not shown). All gap sizes are compatible with the TaTasR structure, so we hypothesize that reduced activity with larger gaps is due to the energetic penalty of breaking more base-pairs than are formed. (For example, tigRNA spacer A/B pairing introduces 18 bp, a +3 gap, which breaks 21 bp.) **(E)** Apo TaTasR protein was loaded with in vitro transcribed and dephosphorylated tigRNA variants as indicated. NT, non-targeting; RC, reverse-complemented. The schematic shows the equivalency of tigRNAs with A-B spacers and tigRNAs with B-A spacers. **(F)** Scheme of *in vitro* TAM identification screen. **(G)** Upstream and downstream TAMs. Top: TaTasR. Bottom: SpTasH.

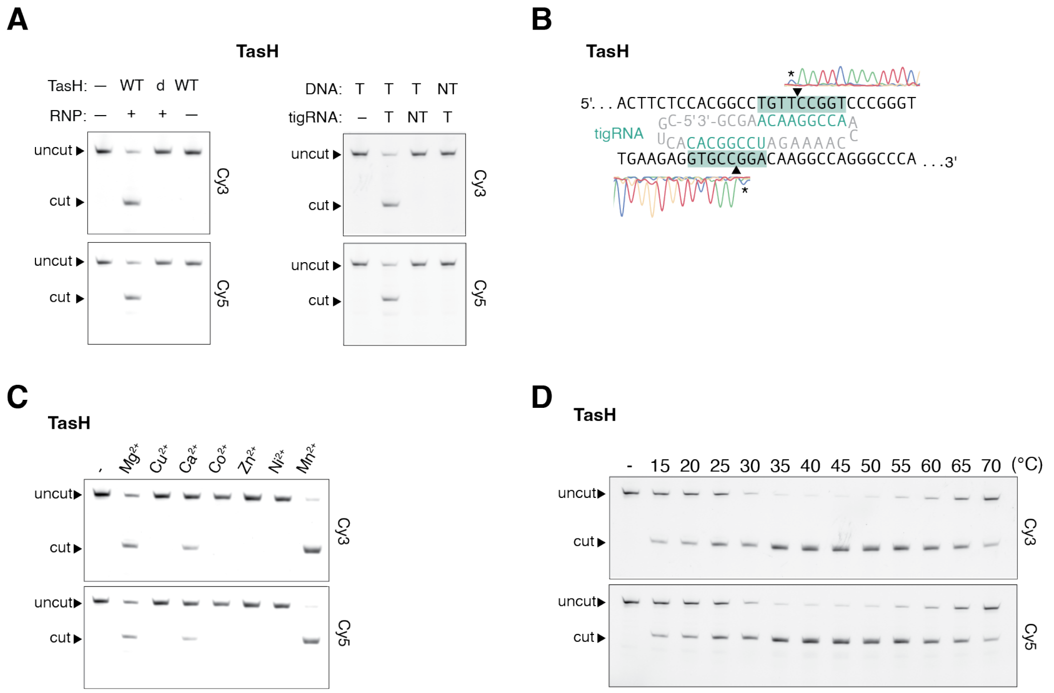

Fig S8. Biochemical properties of SpTasH
(A) In vitro cleavage reactions with TasH. Left: SpTasH (WT) or catalytically inactivated HNH domain (d, H54A/ H58A double mutation) RNPs were purified and incubated with synthetic target DNA matching tigRNA1. (RNP "–" indicates apoprotein was used). Right: Purified WT SpTasH apoprotein was incubated with synthesized tigRNA1, no RNA, or a non-targeting tigRNA (NT), with the target (T) DNA substrate or non-targeted (NT) substrate. tigRNA2 from the TaTasR TIGR array was used as the non-targeting tigRNA, and a PCR amplicon of the SpTasH target was used for the non-targeted DNA substrate. **(B)** Sanger traces for sequencing of the TasH in vitro-cleaved optimized DNA target. The polymerase used in Sanger sequencing adds a non-templated A after running off the template, indicated with an asterisk, and this delineates the precise cleavage site. **(C)** Target dsDNA cleavage by SpTasH in the presence of various divalent metal ions. All subsequent experiments are performed using Mg^{2+}. **(D)** Target dsDNA cleavage by SpTasH at various temperatures.

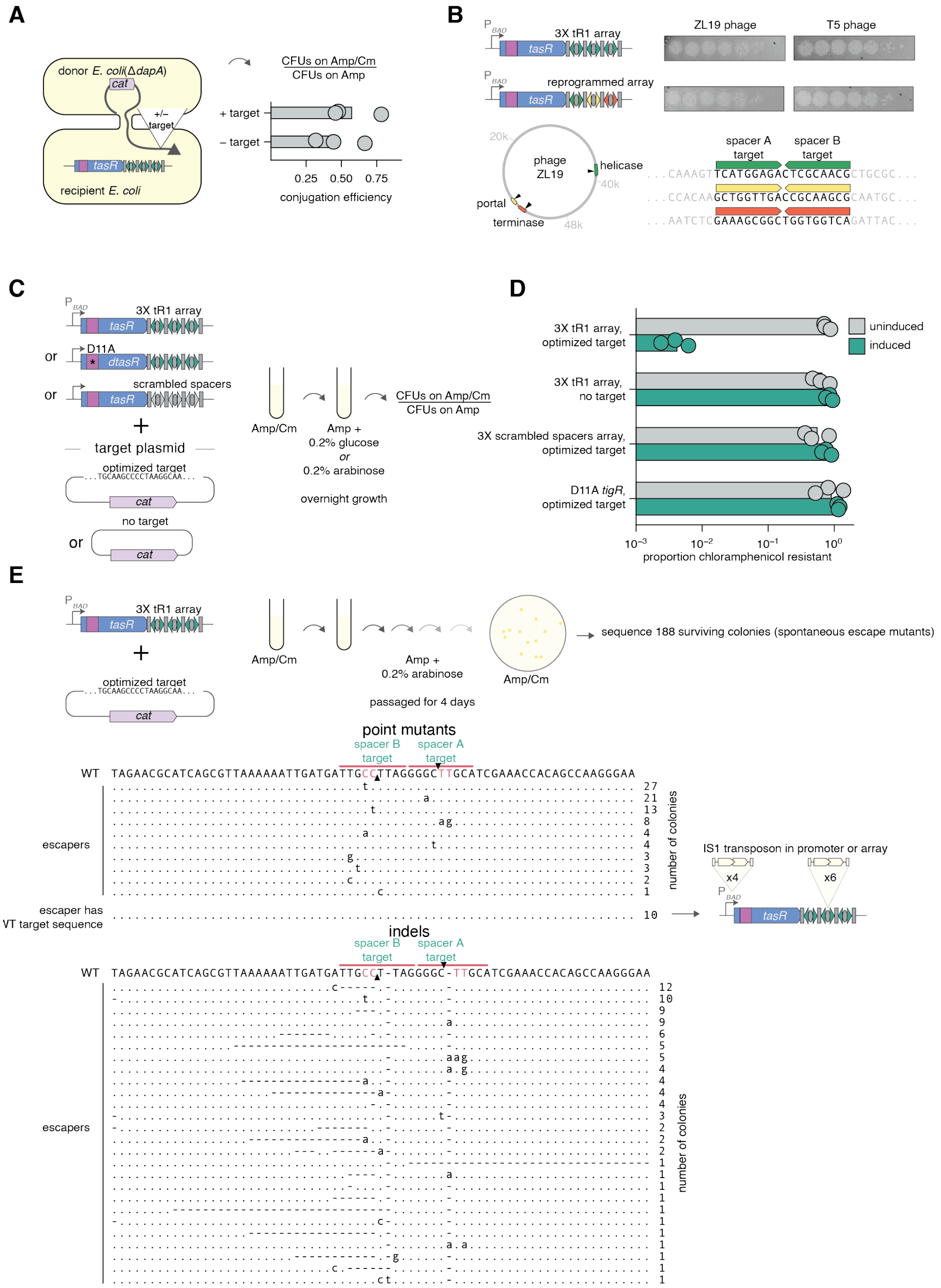

Fig. S9. In vivo activities of TaTasR on plasmid maintenance, conjugation and phage infection

(A) Conjugation efficiency assay. An RP4 oriT-containing plasmid with or without the biochemically optimized target sequence for tigRNA1 was conjugated into a recipient strain containing TaTasR and 3x tigRNA1 (3X tR1). Transconjugants were selected after 3.5 hrs of

growth and conjugation efficiency was determined as indicated. Note that the donor cells cannot grow on LB in the absence of diaminopimelic acid (Δ*dapA* mutant) so are excluded from the CFU calculations. *cat*, chloramphenicol acetyltransferase (resistance marker). **(B)** Phage defense assay. *E. coli* Stbl3 contained a TIGR-TasR plasmid either encoding 3 copies of tigRNA1 (3X tR1), or 3 tigRNAs designed to target different regions of the phage ZL19 genome as indicated. Plaques are shown for 10-fold dilution series of phage ZL19 or T5 on these strains. **(C)** Schematic of a plasmid maintenance assay. *E. coli* was cotransformed with arabinose-inducible TIGR-TasR system variants (on an ampicillin resistance marked plasmid) and a target plasmid with a chloramphenicol-resistance gene marker and the biochemically optimized target sequence for tigRNA1. The system was induced or repressed with arabinose or glucose respectively and cells were grown overnight without chloramphenicol selection. Plasmid maintenance was determined as the number of chloramphenicol-resistant colonies isolated after overnight induction of the TIGR system relative to total ampicillin-resistant colonies. *cat*, chloramphenicol acetyltransferase (resistance marker); Amp, ampicillin; Cm, chloramphenicol. (**D**) Plasmid loss after one night of growth without antibiotic selection. Uninduced cells were grown with 0.2% glucose to repress the P_{BAD} promoter, induced cells were grown with 0.2% arabinose to induce the promoter. Only the induced combination with matching TIGR spacers and target plasmid leads to detectable plasmid loss. (**E**) Selection of escape mutants. Cells were passaged for a further three nights in LB + ampicillin + arabinose without chloramphenicol selection. At this point, plasmid maintenance was estimated as 1 in 10,000 CFUs. Dilutions of cells were plated on media containing ampicillin and chloramphenicol to select for escape mutants, plasmids were prepared and sequenced by NGS. All 38 unique escape mutants are shown aligned to the wild-type targeted plasmid, along with the number of colonies the mutation was found in. Periods indicate matches to the WT target plasmid, lower case letters indicate point mutations, dashes indicate gaps. Mutants are grouped by simple point mutations and larger insertions/deletions (indels). The 2 escape mutants with wild-type target plasmids had IS1 transposon insertions either in the P_{BAD} promoter or TIGR array of the TIGR system plasmid.

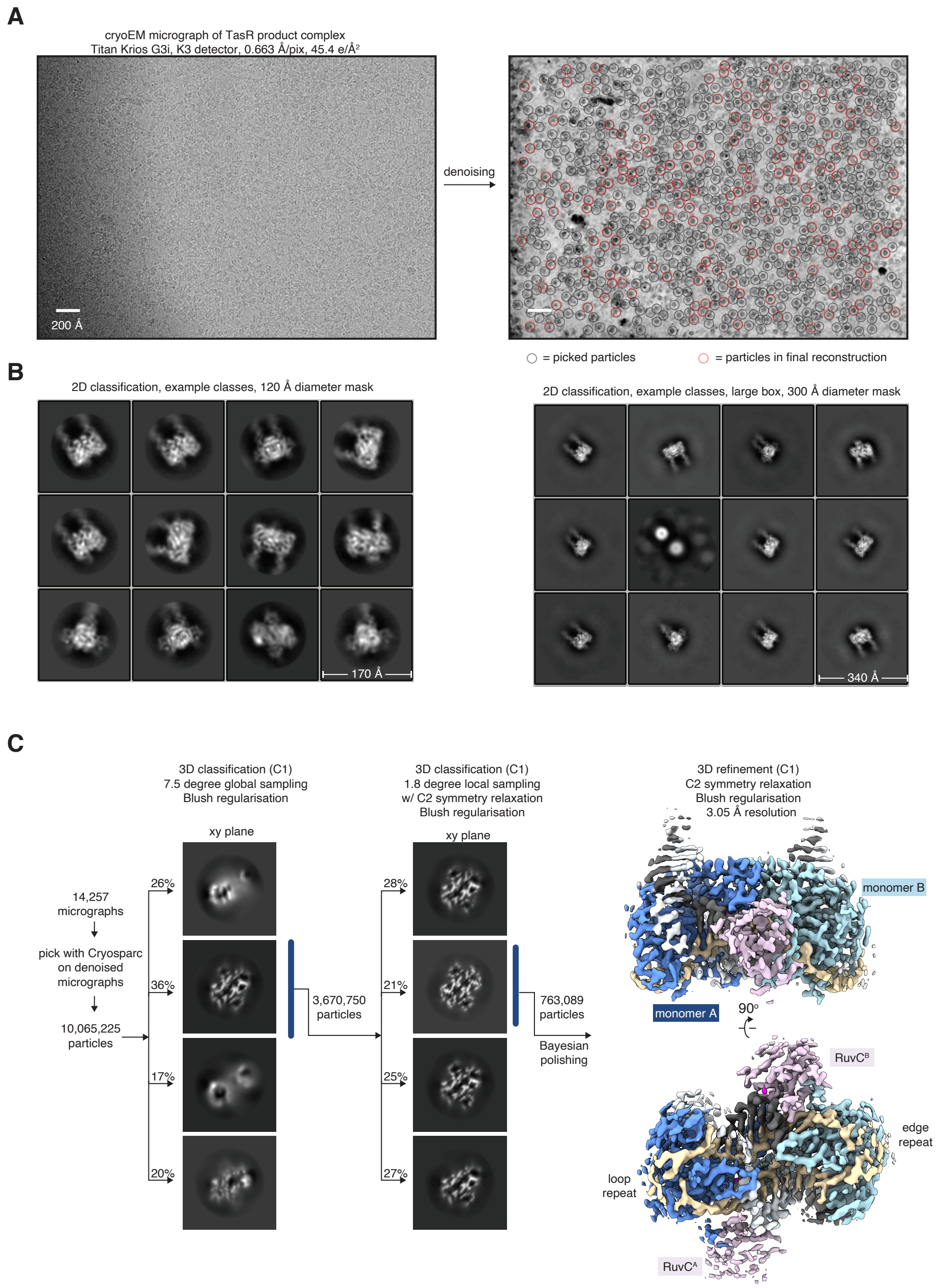

Fig. S10. Cryo-EM data processing

(A) Example cryo-EM micrograph for the TasR RNP + DNA product complex. Left shows the raw micrograph, right shows the micrograph after denoising in cryoSPARC. Denoised micrographs were used for particle picking (grey circles). The particles in the final reconstruction are circled in red. **(B)** Example 2D classes for the particles in the final reconstruction. Right shows some example classes from the same particles but with a larger box and circular mask, showing no evidence for higher-order structure beyond dimerization (e.g. tetramers as in IS110). **(C)** Cryo-EM processing workflow. All steps except particle picking were performed in RELION-5.0. Classes from 3D classification are shown as central slices. The final sharpened map is shown on the right.

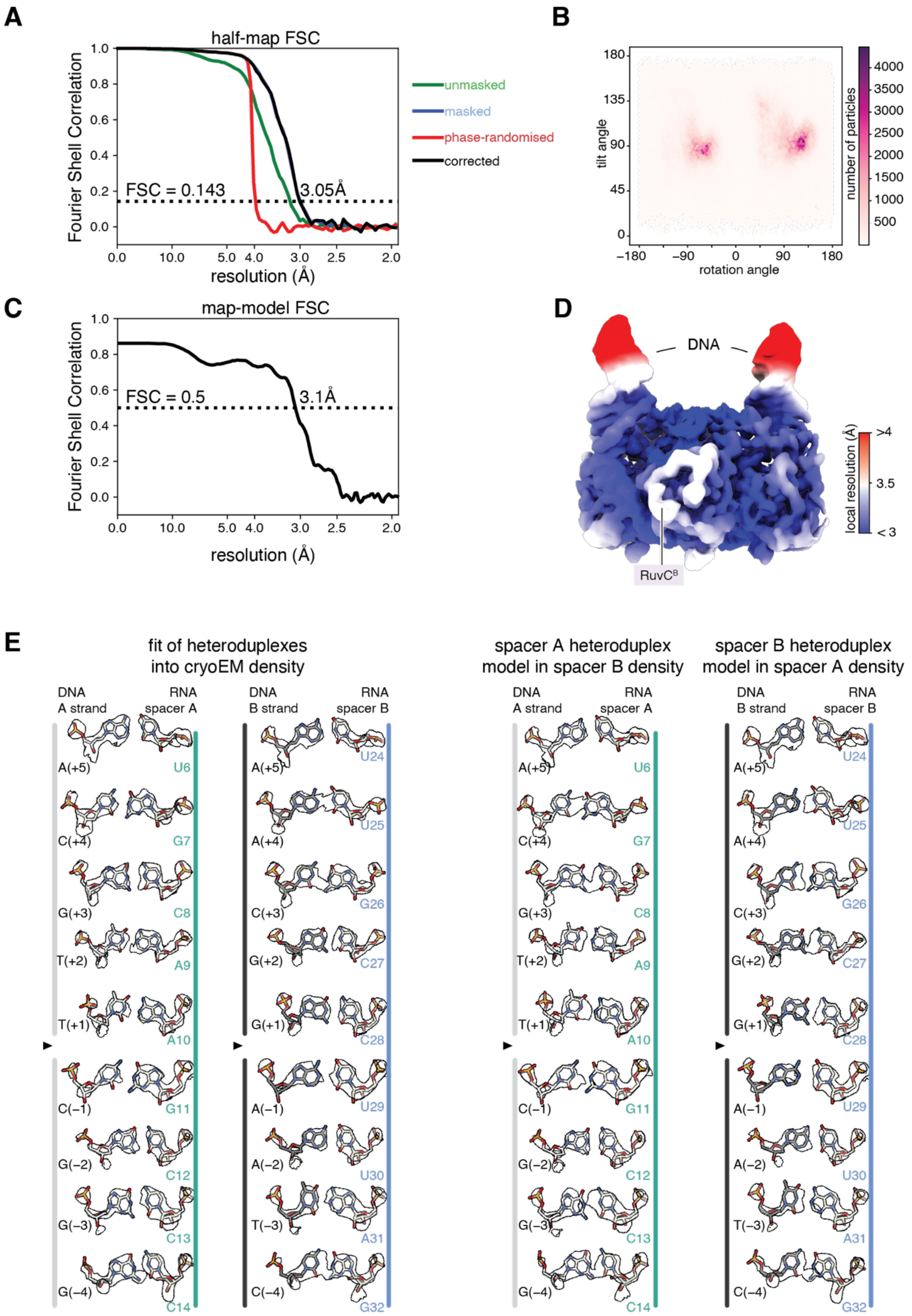

Fig. S11| Cryo-EM density assessment

(A) Gold-standard Fourier Shell Correlation (FSC) curves for the final reconstruction as calculated in RELION. **(B)** Orientation distribution for the final reconstruction. **(C)** Map-model FSC as calculated with Phenix. **(D)** Local resolution of the final map as calculated in RELION. The local resolution-filtered map is shown with an additional low-pass filter applied to visualize the peripheral DNA, which is flexible. **(E)** Cryo-EM density masked around each base pair within the two RNA DNA heteroduplexes. Due to the pseudosymmetry of the complex, if refined with C2 symmetry the densities for the spacer A and spacer B duplexes would look identical. If correctly refined with C1 symmetry, these densities should appear distinct where spacers A and B differ in sequence. The left shows the heteroduplexes in the maps as modelled. The right shows the fit of the spacer A heteroduplex into the spacer B density, and vice versa. In most positions, the distinctly larger density for purines vs pyrimidines allows distinction of spacer A vs spacer B and shows that the pseudosymmetry of the complex was accounted for during cryo-EM reconstruction.

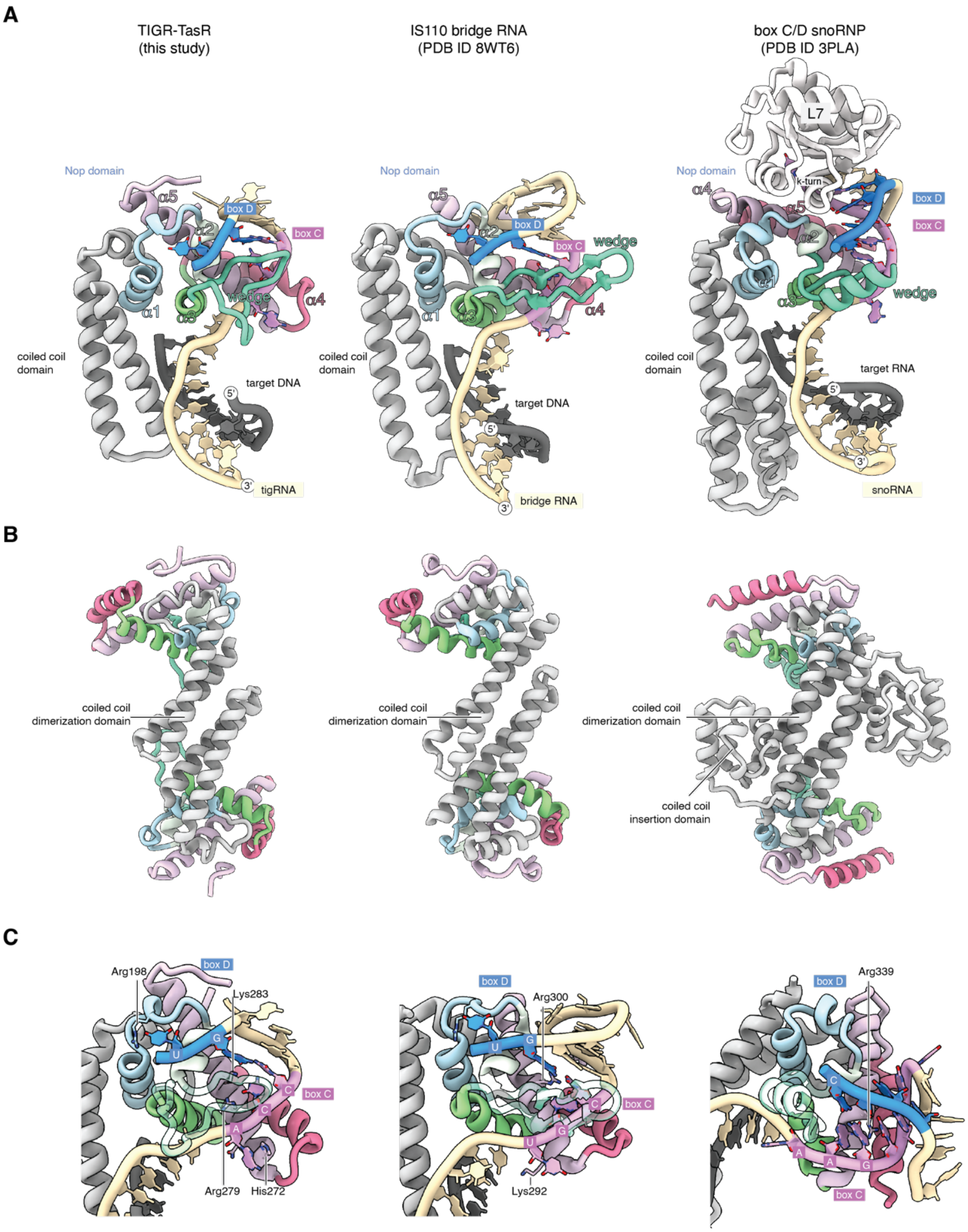

Fig. S12| Structural comparison of TasR with IS110 and box C/D snoRNP
(A) Comparison of the Nop and coiled-coil domain of TasR (this study), the IS110 transposase (*32*), and the box C/D snoRNP Nop5 protein (*111*). One RNA/DNA heteroduplex is shown from each structure and the equivalent box C/D motifs are highlighted. The box C/D snoRNP structure additionally shows the L7 protein (L7Ae archaeal equivalent) recruited to the k-turn formed by box C/D. Equivalent alpha helices are coloured identically. Nop5 in the box C/D snoRNP contains the equivalent to alpha helix 4 in TasR and IS110 after the equivalent to alpha helix 5, so the numbering of these two elements is reversed. **(B)** Comparison of the dimerization

interfaces, which are all identical. **(C)** Comparison of the box C/D motif binding sites on the Nop domain.

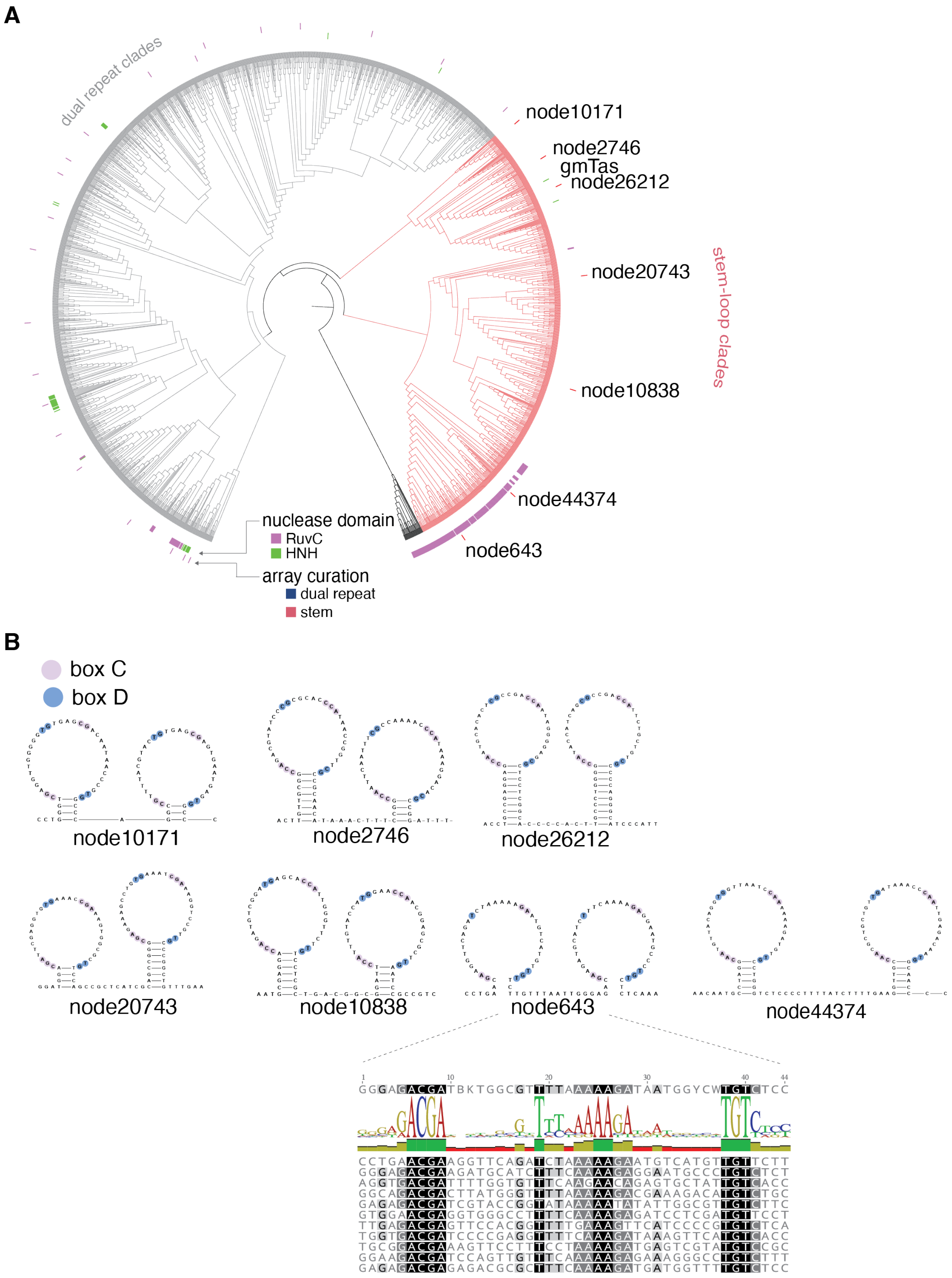

Fig S13| Diversity of Stem TIGR arrays

(A) Phylogenetic tree of the Nop domain in the Tas family. The inner ring indicates the presence and type of nuclease: green for HNH and purple for RuvC. Nodes around the tree correspond to loci manually inspected for stem loop arrays, with associated stem secondary structures shown in

panel **B**. Red branches denote association with stem arrays. Detailed loci coordinates and sequences for each node are provided in table S1. Blue branches represent dual repeat arrays depicted in fig. S4). **(B)** Structural prediction of stem units. Predicted secondary structures of two representative stem units are depicted for loci associated with stem arrays. C and D motifs are highlighted in orange and yellow, respectively. Below, a sequence alignment is provided for a stem-less example for comparison, illustrating the diversity and conservation within the TIGR-associated stem arrays.

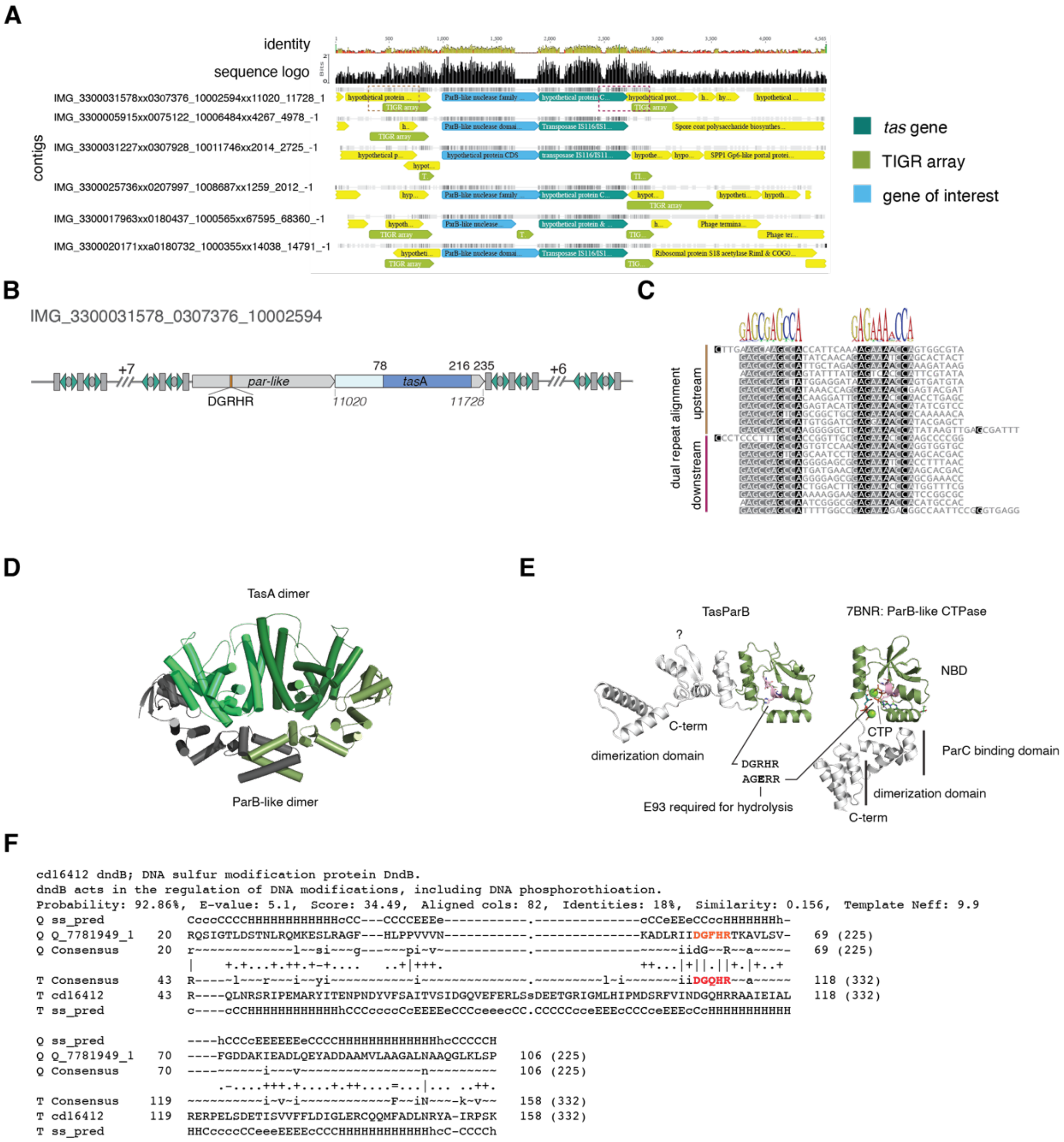

Fig S14| TIGR association with parB-like gene
(A) Locus conservation and variability. Sequence alignment of six loci highlights the conserved operonization of *tasA* (teal color) and *tasParB* (blue color) genes, while flanking regions exhibit variability, suggesting a functional association between the two genes. TIGR arrays are shown in light green and found upstream, downstream, or between *tasA* and *tasParB*. **(B)** Locus organization of TIGR-associated *tasA* and *tasParB* genes. Genomic schematic showing an operon where *tasA* is operonized with a predicted ParB-like domain gene (*tasParB*). The *tasA* gene is colored according to its domain architecture, light blue for the coiled coiled, blue for the RNA binding domain, and grey for C-terminal region. A conserved motif DGRHR is highlighted within *tasParB*. Locus coordinates are provided. The operon is surrounded by 2 TIGR arrays. **(C)** Repeat array alignment. Alignment of upstream and downstream array repeats from the locus in panel **B**. Both arrays feature truncated edge repeats at their terminal positions, appearing at both the beginning and the end of each array. **(D)** AlphaFold2 multimer prediction of *TasA* and

TasParB tetrameric (2xTasA, 2xTasParB) structure. TasA proteins are predicted to form a head-to-head dimer (light and dark green), a feature distinct from other Tas protein family members. TasParB proteins (beige and black) are predicted to dimerize via cofolding of their C-terminal regions, a characteristic also observed in other ParB-like domain systems (*107*). TasParB weakly interacts with TasA. Such prediction suggests more components, e.g. tigRNA and DNA target, might be required to predict the assembly. **(E)** Structural comparison of *TasParB* and ParB-like proteins. Structural comparisons with a known ParB-like CTPase (PDB: 7BNR) revealed similar ParB-like folds (highlighted in green), a conserved dimerization domain in the C-terminal region, and a distinct recruiting domain. In ParB-like CTPases, this domain recruits ParC, but in TasParB, this region is structurally divergent, suggesting a different functional interaction. The catalytic site of ParB-like CTPases is indicated in pink, and mapping these positions onto TasParB revealed a distinct and conserved motif in TasParB, suggesting an alternative enzymatic function for TasParB. **(F)** HHpred analysis of *TasParB*. Sequence-based homology searches using HHpred identify significant similarity between *TasParB* and various ParB-like proteins. Notably, *ParB-like dndB* DNA sulfur modification proteins show similar motifs (TasParB: DGFHR in orange, vs dndB: DGQHR in red) to *TasParB* aligned with catalytic residue positions (see panel **(E)**) suggesting potentially similar enzymatic functionality for TasParB potentially recognizing DNA phosphorothioate modification or play a role in related modifications (*108*, *112*, *113*).

Table S1. (separate file)

Sequence information

This Excel file contains detailed sequence and annotation data for all identified TIGR systems, organized into the following sheets:
(**1**) StudiedSequences. Contains native sequences used for structural mining, and for experimental characterization.
(**2**) ProteinInfo. Lists protein sequences and their genomic coordinates (database, project, contig, start, end, strand) for all TIGR system *tas* genes. Includes node identification numbers (*nodeid50*) used across various supplementary figures and in the phylogenetic tree (data S2). Rep50 indicates genomic coordinates of the representatives (50% of sequence identity clustering) used for the analysis. name indicates the protein name used in the contigs. source indicates the database origins. aa_seq are the protein sequence of the Tas protein.
(**3**) NTD_REP50. Indicates the presence of RuvC or HNH nuclease domains across Tas protein representatives clustered at 50% sequence identity (non-redundant set). Proteins are named according to their nodeid on the phylogenetic tree (data S2).
(**4**) TAX_REP50.Provides taxonomy predictions for representative TIGR-associated contigs (50% sequence identity clusters) using MetaBuli (*91*), and viral origin predictions derived from ViralVerify (*92*) and PhageBoost (*92, 93*). Proteins are named according to their nodeid on the phylogenetic tree (data S2). Protein classifications in column "class_metabuli" are A, archaeal; B, bacterial; V, viral; blank, unclassified.

Supplementary Table 2:
Cryo-EM data collection, refinement, and validation statistics

	TasR RNP in complex with target DNA<br>(PDB 9MTY)<br>(EMDB 48616)
**Data collection and Processing**	
Microscope	Thermo Scientific Titan Krios cryo-TEM
Voltage (keV)	300
Camera	Gatan K3
Magnification	130000
Pixel size at detector (Å/pixel)	0.663
Total electron exposure (e^{-}/Å^2)	45.4
Exposure rate (e-/pixel/sec)	17.82
Number of frames collected during exposure	40
Defocus range (µm)	−1.2 to −2.5
Automation software	EPU
Energy filter slit width	20 eV
Micrographs collected (no.)	14257
Total extracted particles (no.)	10,065,225
Refined final particles (no.)	763,089
Point-group	C1
Estimated error of translations/rotations	0.522 Å / 1.631°
Resolution (global, Å)	
FSC 0.5 (unmasked/masked)	3.71 / 3.32
FSC 0.143 (unmasked/masked)	3.20 / 3.00
Resolution range (local, Å)	2.96 – 5.07 (5th to 95th percentiles within refinement mask)
Map sharpening *B* factor (Å^2)	-115.2
Map sharpening method	relion_postprocess
**Model composition**	
Atoms (non-hydrogen)	7110
Protein residues	586
Ligands	4
RNA/DNA bases	112
**Model Refinement**	
Refinement package	phenix.real_space_refine
- real or reciprocal space	Real space
- resolution cutoff	3.1
Model-Map scores	
-CC	0.73
- Map-model FSC=0.5	3.1 Å
Mean *B* factors (Å^2)	
Protein residues	42.5
Ligands	36.3
RNA/DNA	19.9
R.m.s. deviations from ideal values	
Bond lengths (Å)	0.009
Bond angles (°)	1.311
**Validation**	
MolProbity score	1.17
CaBLAM outliers (%)	0.18
Clashscore	2.43
Poor rotamers (%)	0.39
C-beta deviations (%)	0
EMRinger score	3.83

Nucleic acid geometry	
Correct sugar puckers (%)	94.4
Good backbone conformations (%)	72.2
Ramachandran plot	
Favored (%)	97.2
Outliers (%)	0.4

Table S3. (separate file)
Oligonucleotide and plasmid sequences used in this study

Data S1. (separate file)

Multifasta Sequence Alignment for Custom TIGR HMM Profile
This file contains a curated multifasta sequence alignment derived from the branches mostly lacking RuvC and identified in the phylogenetic tree of hits detected during structural mining. The alignment was further converted into a hmm profile using hmmscan and used for genomic profile mining.

Data S2. (separate file)

Phylogenetic tree of Tas Nop domain
This file contains a Newick-format tree generated from the structural alignment of Tas Nop domain representatives clustered at 50% sequence identity. Each leaf in the tree is annotated with a unique nodeid. These nodeid values correspond to protein entries listed in Supplementary Table 1 for reference to their respective sequences, genomic coordinates, and other associated metadata.
